# Resilient biotic response to long‐term climate change in the Adriatic Sea

**DOI:** 10.1111/gcb.16168

**Published:** 2022-04-12

**Authors:** Daniele Scarponi, Rafał Nawrot, Michele Azzarone, Claudio Pellegrini, Fabiano Gamberi, Fabio Trincardi, Michał Kowalewski

**Affiliations:** ^1^ 9296 Dipartimento di Scienze Biologiche, Geologiche e Ambientali Università di Bologna Bologna Italy; ^2^ 9296 Alma Mater Research Institute on Global Challenges and Climate Change Università di Bologna Bologna Italy; ^3^ Department of Palaeontology University of Vienna Vienna Austria; ^4^ Istituto di Scienze Marine sezione di Bologna Consiglio Nazionale delle Ricerche Bologna Italy; ^5^ Florida Museum of Natural History University of Florida Gainesville Florida USA

**Keywords:** Climate Change, Conservation Paleobiology, Glacial–Interglacial Cycle, Italy, Mediterranean Basin, Mollusk

## Abstract

Preserving adaptive capacities of coastal ecosystems, which are currently facing the ongoing climate warming and a multitude of other anthropogenic impacts, requires an understanding of long‐term biotic dynamics in the context of major environmental shifts prior to human disturbances. We quantified responses of nearshore mollusk assemblages to long‐term climate and sea‐level changes using 223 samples (~71,300 specimens) retrieved from latest Quaternary sediment cores of the Adriatic coastal systems. These cores provide a rare chance to study coastal systems that existed during glacial lowstands. The fossil mollusk record indicates that nearshore assemblages of the penultimate interglacial (Late Pleistocene) shifted in their faunal composition during the subsequent ice age, and then reassembled again with the return of interglacial climate in the Holocene. These shifts point to a climate‐driven habitat filtering modulated by dispersal processes. The resilient, rather than persistent or stochastic, response of the mollusk assemblages to long‐term environmental changes over at least 125 thousand years highlights the historically unprecedented nature of the ongoing anthropogenic stressors (e.g., pollution, eutrophication, bottom trawling, and invasive species) that are currently shifting coastal regions into novel system states far outside the range of natural variability archived in the fossil record.

## Graphical abstract

The latest Quaternary fossil record from sediment cores in the Adriatic coastal systems indicates that nearshore mollusk assemblages of the penultimate interglacial shifted in their faunal composition during the subsequent ice age, and then reassembled again with the return of interglacial climate in the Holocene. These shifts point to a climate‐driven habitat filtering modulated by dispersal processes. The resilient response of the mollusk assemblages to past climate changes suggests that Adriatic nearshore benthic ecosystem may be buffered, to some extent, against future global environmental changes.

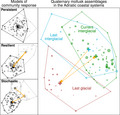

## 1 INTRODUCTION

**FIGURE 1:**
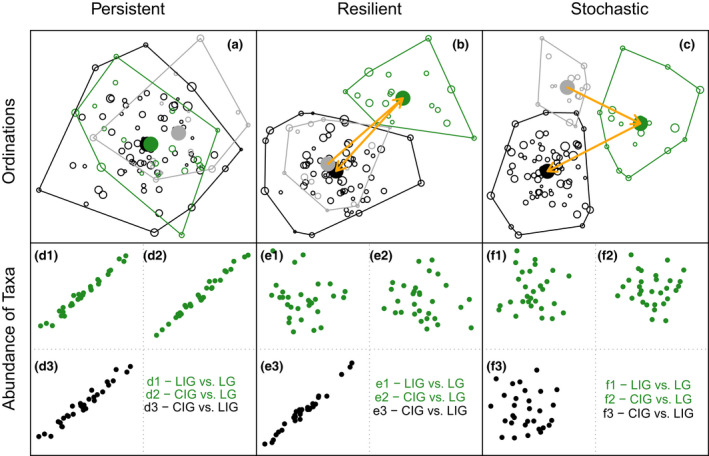
Conceptual framework. Idealized outcomes representing the patterns of community response to glacial–interglacial changes at the regional scale, evaluated by means of ordination analyses (NMDS) and correlation between abundances of species (black: pairwise comparison between the two interglacial units; green: comparisons between glacial and interglacial units). Each column shows one of the three idealized scenarios. Persistent pattern (a and d): Communities maintain species composition and diversity through environmental perturbations even though populations of constituent species shift spatially in concert with sea‐level changes. Resilient pattern (b and e): Communities shift to an altered state during the glacial period but return to previous composition with the re‐establishment of interglacial conditions. Stochastic pattern (c and f) unique species associations characterize communities from all three‐time periods

Predicting the impact of climate change on the structure and composition of biological communities is a major goal of conservation biology (Fredston‐Hermann et al., 2018; Friedman et al., 2020). Simplified models based on thermal tolerances of individual taxa fail to capture the response of communities because they cannot incorporate many other processes that influence species distributions (Doney et al., 2012; Griffith et al., 2018; Steger et al., 2022; Trisos et al., 2020). A long‐term perspective on the variability and resilience of communities is becoming increasingly important, as conservation strategies are faced with accelerating global change (Barnosky et al., 2017). Geobiological archives, such as well‐resolved, fossil‐rich sedimentary successions, can extend the records of ecosystem responses to climatic shifts far beyond the limited timescales of direct ecological monitoring typically restricted to the most recent decades (e.g., Dillon et al., 2020; Harnik et al., 2012; Kidwell, 2015; Tomašových et al., 2020). In particular, the late Quaternary geological record, which archives repeated landward–seaward migrations of coastal environments during glacio‐eustatic cycles, can potentially provide direct documentation of long‐term dynamics of marine ecosystems. These natural experiments allow for contrasting empirical patterns against conceptual models of community response (Figure 1). For example, a community structure can exhibit persistence (resistance sensu Grimm & Wissel, 1997), if it continues through the perturbation without rearranging into a different state (Figure 1a,d; see also Davies et al., 2018; Grime et al., 2008; Hyman et al., 2019). Alternatively, the reorganization of communities can indicate resilience (also called engineering resilience), if a community shifts to an alternate state after perturbation but then reassembles (Figure 1b,e; see also Davies et al., 2018; Nikanorov & Sukhorukov, 2008; O’Leary et al., 2017 and references therein). Finally, communities during intervals of climate change can display highly variable composition resulting from the stochastic processes of ecological drift and individualistic responses of species (stochastic pattern in Figure 1c,f) that can lead to novel or no‐analog communities (Graham et al., 2014; Slišković et al., 2021).

Our understanding of long‐term community dynamics in shallow‐marine environments during the late Quaternary climate oscillations is mostly based on fossil assemblages representing sea‐level highstands associated with warm interstadial and interglacial periods (e.g., Davies et al., 2018; Kowalewski et al., 2015; Martinelli et al., 2017; Pandolfi, 1996). In contrast, few studies have explicitly investigated marine faunal dynamics in comparable depositional environments under both glacial and interglacial conditions (e.g., Aronson & Precht, 2016; Kitamura et al., 2000; Tager et al., 2010), even though such data are necessary for distinguishing between alternative models of community change. In this study, we describe the structure of mollusk benthic assemblages (bivalves, gastropods, and scaphopods) populating shallow, fluvially influenced marine systems during three specific time intervals: (1) the penultimate interglacial (between ~125 and 110 kyr), (2) the subsequent last glacial (between ~18 and 12 kyr cal BP), and (3) the Holocene interglacial (between ~7 kyr cal BP and pre‐1750 CE). This approach allows for tracking the dynamics of faunal assemblages from analogous depositional settings, but during different climate and sea‐level states thus providing a historical perspective on biotic response to long‐term climate change. Here, we used the latest Quaternary fossil record of the Adriatic coastal systems (Text S1; Table S1; Appendices S1–S2) to evaluate if shallow‐marine mollusk assemblages display a persistent, resilient, or stochastic pattern (Figure 1) when responding to major climatic and sea‐level shifts over the last ~125 kyr (Figure S1).

## 2 MATERIALS AND METHODS

The fossiliferous deposits of both interglacial periods are preserved in the subsurface of the present‐day Po coastal plain. In contrast, those of the last glacial period are located more than 250 km southeast of the studied interglacial deposits in the central and southern Adriatic, at the edge of the Mid‐Adriatic Deep, where the shoreline was located during the last sea‐level lowstand (see Text S1; Figure S1).

### 2.1 Data selection criteria

Distribution and preservation of macrobenthic remains in sedimentary successions representing coastal habitats are controlled by a multitude of environmental parameters and sedimentary processes (e.g., Nawrot et al., 2018; Rakocinski et al., 1991). To ensure comparability in terms of environmental context, sedimentation rates, and taphonomic regime, we restricted the analyses to samples from aggrading–prograding lower shoreface to foreshore sedimentary bodies characterized by varying degrees of fluvial influence (hereafter referred as nearshore; Figure S2). This environmental classification of samples was mainly based on previously published sedimentological and micropaleontological inferences and was thus independent from the composition of the mollusk assemblages (see Table S2 for environmental and chronostratigraphic information). The samples (0.150–0.375 dm^3^ each; further details in Appendices S1–S2) were wet‐sieved with 1 mm screen and the remains identified to species level whenever possible. To account for disarticulation of bivalves, the number of isolated valves was divided by two. Multiple ecological descriptors of the studied assemblages (species dominance, sample‐standardized richness, relative abundance, and occurrence frequency), present‐day biogeographic distribution of constituent species (data after Poppe & Goto, 1991, 1993), and multivariate methods were used to compare samples representing the three selected time intervals (i.e., last interglacial—LIG, last late glacial—LG, and current interglacial—CIG; Tables S1–S2). The results were compared to a conceptual framework depicting possible patterns of community change across a glacial–interglacial cycle (persistent, resilient, and stochastic pattern; Figure 1). A comparative assessment of ecological dynamics encompassing the entire land‐to‐deep‐sea depositional profile is not possible due to lack of preservation or limited sampling of different segments of the bathymetric gradient. Freshwater/terrestrial species occasionally recovered in the targeted samples were excluded from the analyses. The dataset for multivariate analyses was further restricted to samples with at least 25 specimens. To check the sensitivity of the results, a more conservative sample size threshold of 60 specimens was also used.

### 2.2 Sample bathymetric estimates

We obtained estimates of the bathymetric distribution of extant species from the Italian mollusk census database (Bedulli et al., 1984). The Italian mollusk database reports, among others, water depth (meters), and specimen abundance (tallied separately for live and dead individuals) for most common mollusk species thriving along the Italian Peninsula. We used these data to estimate preferred water depth for species commonly found in the cored sediments. For those species, its preferred bathymetry was estimated as the abundance‐weighted average depth. Then water depth estimate for each sample was computed by the mean preferred depth of the species found in a sample weighted by their specimen abundances (Wittmer et al., 2014).

### 2.3 Multivariate analyses

Prior to multivariate analyses, the species occurring in one sample only were removed. Subsequently, the sample‐by‐species matrix was converted to relative abundances and fourth root transformed to reduce the effect of hyperabundant taxa. Other commonly used transformation and standardization techniques (e.g., log‐transformation, Wisconsin double‐relativization) produced comparable ordination outcomes (Figure S3; Table S3).

The indirect ordination was performed by nonmetric multidimensional scaling (NMDS) using Bray–Curtis (BC) distance measure (*k* = 2 dimensions). Permutation‐based multivariate analysis of variance (PERMANOVA) based on the same distance matrix was employed to evaluate differences in the locations of the multivariate groups of samples from the three compared time intervals.

### 2.4 Comparison of assemblage composition and model testing

Pairwise comparisons of samples using BC dissimilarity were employed to assess the resemblance between nearshore assemblages from the three periods (i.e., LIG, LG, and CIG). In addition, the observed mean BC distance for each of the comparisons was contrasted against a sampling distribution of means obtained by randomization (based on 1000 iterations) under the null hypothesis that the samples came from the same system. For each pairwise comparison, the randomization procedure involved pooling all specimens and then randomly reassigning them to one of the three time intervals while maintaining the sampling structure of the actual data. For each of the 1000 randomized iterations, mean BC distance was computed and added to the resulting resampling distribution predicted under the null hypothesis.

A similar approach was used in the pairwise evaluation of total species abundances obtained by pooling all samples within each of the three examined time intervals. Each of the three pairwise comparisons (i.e., LIG vs. LG, LIG vs. CIG, LG vs. CIG) was contrasted against a randomized data permutation model depicting a homogenous system based on the pooled species abundances for data combined across all compared time intervals. For each pairwise comparison, specimens were sampled from the pooled species distribution into the sample structures (i.e. the same number of samples and sample sizes as observed) of the compared time intervals. The simulation was repeated 1000 times. For each of the three pairwise comparisons, the resulting 1000 pairs of abundance values (one of each of the two compared time intervals) were obtained for each of the species considered. The modeled distributions of species abundances, predicted under the null hypothesis that samples came from a single underlying species abundance, were plotted together with the observed values.

### 2.5 Bivariate analyses

Spearman's rank correlation coefficient was used to measure the strength of correlation between NMDS sample scores and sample‐standardized species richness (rarefied to 25 and 60 specimens), biogeographic affinity (relative abundance of Mediterranean‐to‐Lusitanian and West African species in each sample), and sample water depth estimates. Lastly, we used information on the present‐day biogeographic distribution of the species as an indicator of their climatic affinity to better understand the relationship between shifts in species composition and paleoclimatic changes (Figure S1). In this approach, relative abundances of species grouped according to their current biogeographic distributions were plotted to evaluate changes in the biogeographic and climatic affinity of the macrofaunal stock across glacial–interglacial transitions.

### 2.6 Software and data access

Specific details on the parameters and bivariate and multivariate statistical test and procedures implemented in this study are given in the captions of figures, tables, and relevant supplementary online materials. All analyses were performed in R (R Development Team, 2018, v 4.0.5) and Excel. The “vegan” package (Oksanen et al., 2018) was used to carry out ordinations and PERMANOVA. Resampling models were written using standard base functions available in R. Codes and data are provided in the supporting information.

## 3 RESULTS

**FIGURE 2:**
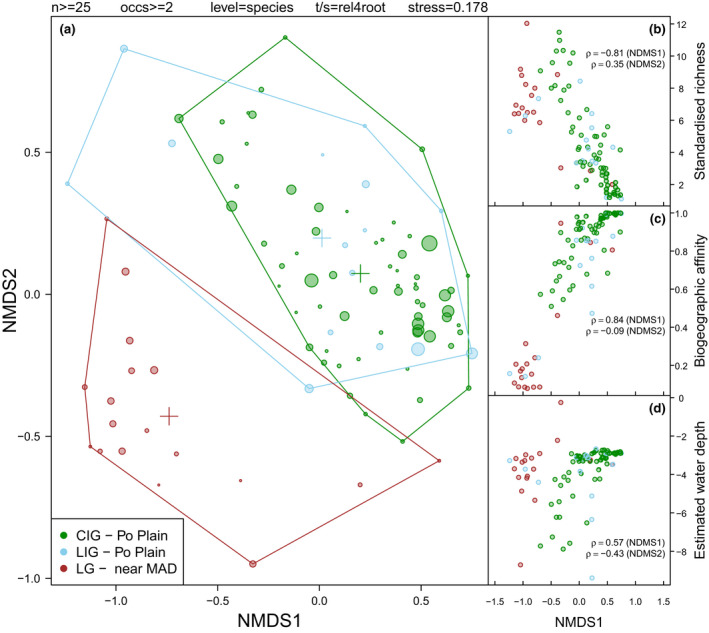
Gradient and rank correlation analyses: (a) NMDS ordination of nearshore samples containing at least 25 specimens (see also Figure S5 for an NMDS output based on sample size threshold of 60 specimens). Relative abundance of species was fourth root transformed. Samples are color‐coded according to the climatic interval: green—current interglacial (CIG), light blue—last interglacial (LIG), and dark red—last late glacial (LG). The size of each point is proportional to sample size. Convex hulls delimit the ordination space occupied by each group of samples. (b) Correlation between NMDS axis 1 sample scores (NMDS1) and species richness rarefied to 25 specimens. Standardized species richness for relatively small samples tends to be primarily driven by evenness, so the two measures are strongly correlated. (c) Correlation between NMDS1 and relative abundance of Mediterranean‐to‐Lusitanian and West African species recovered in each sample. (d) Correlation between NMDS1 and the sample water depth estimates based on species bathymetric preferences (see Materials and Methods for details). In b–d panels, rank correlation coefficient ρ is shown also for NMDS axis 2 sample scores

To evaluate macrobenthic assembly dynamics during climatic shifts, we used 223 nearshore samples from 18 stratigraphically well‐constrained sediment cores (Appendix S1). The samples yielded cumulatively 113 species and 71,282 fossil specimens subdivided into three datasets: 21 LIG samples including 11,413 fossils and 45 species, 32 LG samples including 3381 fossils and 60 species, and 170 CIG samples including 56,488 fossils and 78 species (Appendices S1–S2; Table S1). To develop cross‐validation assessments, we contrasted the results with outcomes of empirically calibrated resampling models simulating patterns expected under the null hypothesis that the recovered assemblages originated from the same regional pool of species (see model testing in Materials and Methods). In the NMDS ordination projection, CIG and LIG sample groups overlapped strongly, whereas LG samples plotted separately (Figure 2a). NMDS axis 1 scores were negatively correlated with sample‐standardized diversity estimates (Spearman's rank correlation ρ = −.81, *p* < .001; Figure 2b) and positively correlated with the proportion of Lusitanian specimens (ρ = .84, *p* < .001; Figure 2c), defined as those specimens that belonged to species for which the present‐day geographic ranges do not extend northward beyond the warm‐temperate Lusitanian province. In addition, quantitative bathymetric estimates based on faunal composition were highly congruent with the independently derived estimates of water depth (Figure S2; Table S2), confirming that all sampled assemblages represented shallow‐water (<10 m) habitats (Figure 2d). These results suggest that LG samples represented habitats and water depths comparable to those of the LIG and CIG interglacial samples but were characterized by higher species richness and depressed abundance of exclusively Mediterranean‐to‐Lusitanian species when compared to the interglacial samples (Figure 2b–d, S4). In contrast, the interglacial samples were strongly dominated by *Lentidium mediterraneum—*an infaunal filter feeder, representing more than 85% of specimens in both interglacial groups of samples (Table S4).

**FIGURE 3:**
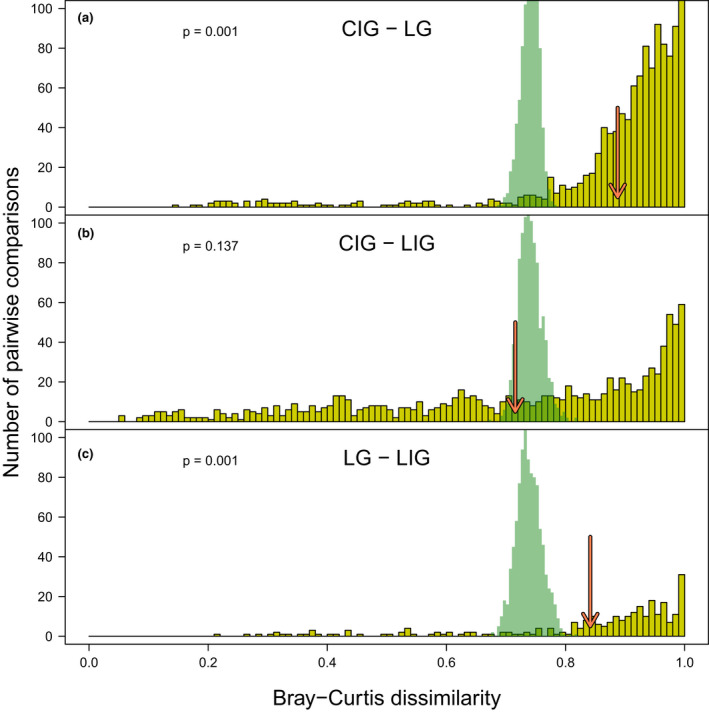
Distribution of pairwise Bray–Curtis (BC) distances between samples representing glacial and interglacial assemblages. (a) Current interglacial and last late glacial (CIG‐LG, based on 1170 pairs of compared samples). (b) Current interglacial and last interglacial (CIG‐LIG based upon 975 pairs of compared samples). (c) Last late glacial and last interglacial (LG‐LIG based on 270 pairs of compared samples). Red arrows mark the location of the observed mean values BC distances for each frequency distribution of the three pairwise comparisons. The x‐axis reports BC dissimilarity range, zero value indicates that two samples have the same faunal composition, one no species in common. In green sampling distributions of means based on randomization (based on 1000 iterations), under the null model that the samples came from the same system. Pairwise comparisons are based on the same species relative abundance matrix as the one used for the NMDS (*n* ≥ 25 specimens and rare species removed)

**FIGURE 4:**
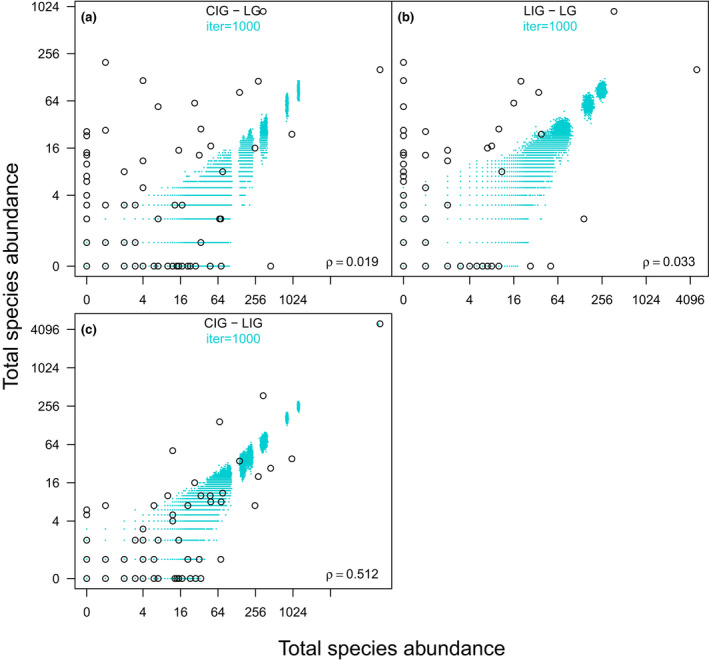
Pairwise comparisons of species total abundances (total counts in pooled data from each interval). (a) Current interglacial and last late glacial (CIG‐LG, upper left panel, x‐axis: CIG, y‐axis: LG). (b) Pleistocene interglacial and last late glacial (LIG‐LG, upper right panel, x‐axis: LIG, y‐axis: LG). (c) Holocene and Pleistocene interglacials (CIG‐LIG, lower panel, x‐axis: CIG, y‐axis: LIG). Species total abundances have been log‐transformed. The output of the randomization model based on 1000 iterations highlights the portion of two‐dimensional space in which the points should fall under the null model of a homogenous system. Spearman's rank correlation (ρ) for each pairwise comparison is reported on each panel; it is significant only for the interglacial pairwise comparison (i.e., CIG‐LIG, *p *< .001; see also Table S6)

Permutational multivariate analysis of variance (PERMANOVA) provided further evidence for the distinct species composition of the LG assemblages and strong similarities between the two interglacials (Table S5). However, PERMANOVA results can be sensitive to the unbalanced sampling design (Anderson & Walsh, 2013). Therefore, we also compared the observed BC dissimilarities between individual samples from different time intervals with the predictions of the resampling models (Figure 3a,b). Only in the LIG versus CIG comparison, the observed mean pairwise BC dissimilarity fell within the sampling distribution of means expected if the samples from the two interglacial periods came from a species pool with a homogenous composition and comparable abundance structure (Figure 3b). In contrast, the average dissimilarity between LG samples and samples from either of the studied interglacials departed significantly from the null model predictions and was much higher than the observed mean pairwise distance between LIG and CIG samples (Figure 3a,c; *p* = .001). Moreover, when individual samples were pooled together in each time interval (Figure 4a–c), the two interglacials were also characterized by a very similar species abundance structure, with a positive Spearman's rank correlation (ρ = .51; *p* < .001, Figure 4c and Table S6). Species abundances in LG and either of the interglacials were not significantly correlated (ρ < .035 and *p* > .70 in both cases; Figure 4a,b and Table S6). Lastly, a comparable stock of species dominated the Adriatic nearshore settings during both interglacials (Table 1), with seven of the most dominant species recovered from the CIG interval also belonging to the top 10 species in the LIG samples (Table S4). LG group of samples shared only four of the top 10 most abundant species with the CIG (Table 1, Table S4).

**TABLE 1:** The 10 most abundant species in the current interglacial—CIG (pre‐modern Era), and their ranking in the other two time periods (Pleistocene last late glacial—LG and Late Pleistocene interglacial—LIG)

Species (Total number of species = 113)	Authorship	CIG	LG	LIG
*Lentidium mediterraneum*	(O.G. Costa, 1830)	1	3	1
*Chamelea gallina*	(Linnaeus, 1758)	2	12	5
*Donax semistriatus*	Poli, 1795	3	absent	7
*Spisula subtruncata*	(da Costa, 1778)	4	1	2
*Bittium reticulatum*	(da Costa, 1778)	5	5	8
*Varicorbula gibba*	(Olivi, 1792)	6	15	16
*Ecrobia* gr. *ventrosa^1^*	(Montagu, 1803)	7	6	6
*Bela formica^2^*	(Nordsieck, 1977)	8	23	10
*Peronidia albicans*	(Gmelin, 1791)	9	absent	14
*Tritia varicosa^3^*	(W. Turton, 1825)	10	41	32

Note

Taxonomic notes: 1 This is a group of very similar and highly variable species: *Ecrobia ventrosa*, *Hydrobia acuta* and *Eupaludestrina stagnorum* not easily distinguishable by the shell features; 2 *Bela formica* is considered taxon inquirendum previously synonymized with *Bela nebula*; 3 commonly reported as *Tritia pygmaea* (Lamarck) a junior secondary homonym of *Muricites pygmaeus* Schlotheim.

**FIGURE 5:**
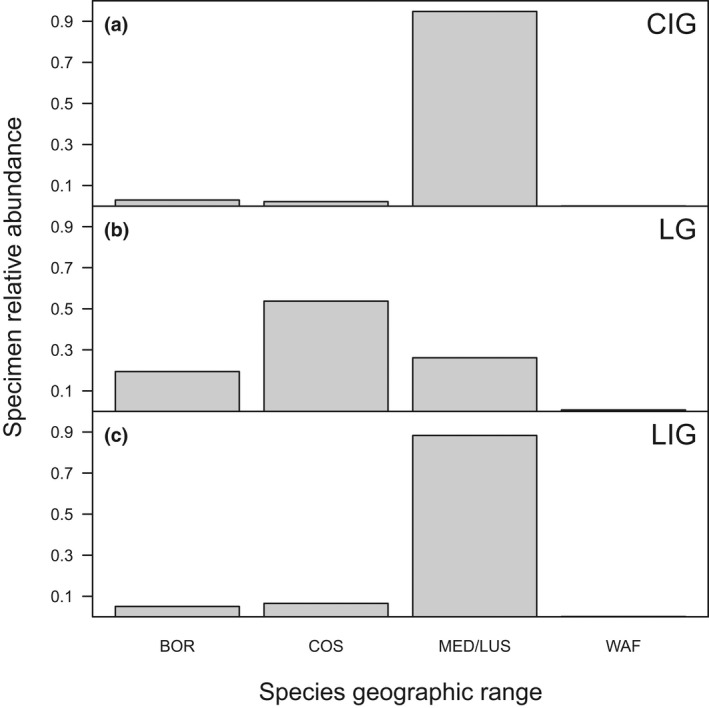
Comparisons of species total relative abundances grouped according to their biogeographic distribution. (a) Current interglacial—CIG; (b) last late‐glacial—LG; (c) last interglacial—LIG. Information on the geographic range of mollusk species is after Poppe and Goto (1991, 1993). Abbreviations for biogeographic affinity of species distribution: BOR = species occurring in the Mediterranean, Lusitanian, and Boreal provinces; COS = species of cosmopolitan distribution (i.e., occurring from West African until Boreal provinces); MED/LUS = species occurring in the Mediterranean and/or Lusitanian provinces; WAF = species occurring in the Mediterranean, Lusitanian, and West African provinces

Relative abundances of species with different biogeographic affinities (Figure 5) were comparable between the two interglacials, but differed from those observed in the LG. Specifically, the LIG and CIG samples were dominated by species restricted to Mediterranean and Lusitanian provinces (>88% of specimens; Figure 5a,c). The relative abundance of this group decreased down to 26% during the LG period. In contrast, cosmopolitan species, today occurring in both (sub)tropical and cold‐temperate East Atlantic regions, increased in relative abundance from less than 7% in both interglacials to 54% in the LG period. The LG samples are also characterized by a higher relative abundance (19%; Figure 5b) of Boreal species (ranging from the Mediterranean to the cold‐temperate NE Atlantic), compared to the interglacial samples (5% and 3% in the LIG and CIG, respectively).

## 4 DISCUSSION

### 4.1 Nearshore biotic response to glacial–interglacial cycles

The macrobenthic assemblages from the two interglacials are statistically indistinguishable in terms of species composition (Figures 2, 3, 4, 5; Table 1) and characterized by high dominance and low richness both at the scale of individual samples (Figure 2b, Figure S4) and the regional species pool (Figure S6; Table 1, Table S4). However, they remain distinct from more species‐rich glacial assemblages representing similar shallow‐marine habitats. These results indicate that Late Pleistocene interglacial nearshore associations of the Adriatic transitioned to a different state during the last glacial period, but when interglacial climatic conditions were reestablished in the Holocene, these mollusk associations shifted back to the species composition and abundance structures characteristic of the previous interglacial. Minor differences between the current and previous interglacial assemblages suggested by the ordination analysis (Figure 2a) are likely driven by sampling effects (see NMDS results limited to larger samples only; Figure S5). Despite high spatial and temporal variability of deltaic habitats, the similarity of the two interglacial assemblages suggests that large‐scale, long‐term environmental drivers overwhelmed local effects of changing coastal physiography or distance to the river or distributary channel mouths. Overall, the observed paleoecological pattern of nearshore assemblages is consistent with the resilient model of long‐term community response to glacial–interglacial climate and sea‐level cycles (Figure 1a,d).

The observed resilient response of mollusk assemblages from dynamic, fluvially influenced nearshore settings (McKinney, 2007) is also consistent with patterns observed in other marine systems. Deep‐sea benthic foraminiferal assemblages of the Santa Barbara Basin (USA) exhibited a similar repetitive faunal turnover in response to millennial‐scale variations in oxygen concentrations related to Dansgaard–Oeschger climatic cycles (Cannariato et al., 1999). Pleistocene coral reefs of Papua New Guinea were characterized by recurring coral associations during sea‐level highstands and compositionally distinct lowstand assemblages over the past 416 kyr (Pandolfi, 1996; Tager et al., 2010). Interestingly, the variable composition of lowstand coral associations contrasts with the persistence of microbenthic and calcareous algal assemblages from the same reef ecosystem (Tager et al., 2010). Finally, the resilient response of onshore macrobenthic associations together with higher turnover in offshore environments was documented in the deep‐time fossil record during higher order sea‐level fluctuations over millions of years (Danise & Holland, 2017). Some late Cenozoic marine mollusk faunas underwent continuous gradual changes in species composition during past climate oscillations, in spite of cyclic recurrence of similar environments (Stanton & Dodd, 1997). Such a pattern is similar to the substantial shifts in plant and vertebrate communities frequently observed in Quaternary terrestrial ecosystems, which have been linked to differential responses of individual species to highly dynamic environmental changes (Jackson & Blois, 2015). Thus, rather not surprisingly, biotic responses to naturally occurring climate changes during the Quaternary appear to have varied greatly across ecosystem types and organismal groups.

Taken together, the results of this and previous studies suggest that resilient patterns can be scale invariant, and more prevalent in communities that inhabit environmentally unstable habitats and may thus be preadapted to cope with long‐term climate and sea‐level changes. Indeed, the studied nearshore system is dominated by r‐selective eurythermal species capable of rapid recolonization whenever favorable environmental conditions return. In addition, a large suite of more vulnerable (i.e., less thermally tolerant) Pliocene Mediterranean taxa had been previously extirpated in a series of regional extinctions (Monegatti & Raffi, 2001). Therefore, the impact of the Quaternary climate shifts has been attenuated in the Mediterranean Sea by a long history of major climatic fluctuations that had shaped the regional pool of taxa in this region.

### 4.2 Mechanisms of change and ecosystem resilience

Understanding how the structure and composition of past ecosystem change through time allows us to depict hypothetical scenarios of community dynamics in the face of climate change. Broad models of community assembly fall within three categories: interaction assembly, environment assembly, and neutral assembly (Vellend, 2016 and references therein). Interaction assembly model considers communities structured primarily by ecological locking among species due to strong interspecific interactions (e.g., predation or resource competition), resulting in limited membership. Environment assembly model regards community membership principally as the result of deterministic species responses to the changing physical environment. Finally, communities structured by stochastic (neutral) processes have no membership constraints, strong hysteresis, and high variability under comparable environmental conditions.

Before we assess those three models of community assembly, we should first note that in the semi‐enclosed Adriatic basin, the transitions from interglacial to glacial periods were characterized by changes in the basin morphology, sea surface temperature, salinity, and circulation pattern (Maselli et al., 2014; Piva et al., 2008, Figure S1). During the last glacial interval, the targeted portion of the Adriatic experienced high sedimentation rates, eutrophic waters, and frequent freshwater inflows (Asioli et al., 2001; Pellegrini et al., 2018). Although similar conditions were present also during the middle‐late Holocene (Amorosi et al., 2016; Pellegrini et al., 2021), some of the key abiotic factors are estimated to have differed strongly between glacial and interglacial periods. Salinity was lower during the LG period due to a more confined Adriatic basin and higher inflow of freshwater from the Po River (Asioli et al., 2001; Pellegrini et al., 2017). Moreover, the estimated sea surface temperatures (SSTs) were ~6°C lower during the Last Glacial Maximum (LGM) compared to the Holocene climatic optimum (Capotondi, 2004; but see also Piva et al., 2008), an offset slightly lower than that estimated for the Adriatic between the LGM and LIG (Hoffman et al., 2017, see also discussion below).

Shifts in the relative abundance of species with different biogeographic and climatic affinities (Figures 2c, 5; Table S4) in targeted nearshore assemblages follow these environmental changes. Samples from all three time intervals were dominated by mollusks that thrive in shallow‐water habitats with fine sand substrates in the modern Mediterranean Sea (Pérès & Picard, 1964). However, during LIG and CIG, species that today are restricted to subtropical to warm‐temperate Mediterranean and Lusitanian provinces had much higher relative abundances (i.e., *L*. *mediterraneum*, *Chamelea gallina*, and *Donax semistriatus*). Assemblages from LG were characterized by higher richness and evenness and were dominated by species whose present‐day biogeographic ranges extend farther northward into cool‐temperate regions of the Eastern Atlantic (e.g., *Spisula subtruncata*, *Fabulina fabula*; Petersen, 1913). Notwithstanding the different composition and diversity structure, glacial and interglacial nearshore communities all share eurytopic species that thrive in fluvially influenced settings along an onshore–offshore gradient, such as *Ecrobia ventrosa* species complex and *Varicorbula gibba*.

This biotic pattern is consistent with the regional paleotemperature record (Figure S1d; Capotondi, 2004; Piva et al., 2008) and suggests that nearshore Adriatic mollusk communities most likely followed the environmental assembly model, where community composition is largely determined by the overlap between their environmental tolerances and the local environmental conditions (Jackson & Blois, 2015). Thus, in LG, the dominance of cosmopolitan taxa characterized by broad habitat niches and thermal tolerance (so expected to be found across heterogeneous environments and more resistant to thermal stresses) suggests the predominant role of environmental filtering (species sorting) in driving the shifts in the assemblage composition rather than biotic perturbation related to species interactions expected during community coalescence (blending of distinct communities) (Rocca et al., 2020). During the last glacial period, lower temperatures limited the fitness of a subset of r‐selected nearshore species that are characterized by explosive population dynamics and can reach high densities in favorable conditions but are less adapted to a colder climate (e.g., *L*. *mediterraneum*, *C*. *gallina*; Figure 5b; Table 1, Table S4). Consequently, their abundance and occurrences in the northern regions of the Mediterranean, including the Adriatic Sea, were greatly reduced, in some cases limiting their distribution to the southern coasts of the basin. Species characterized by broader thermal tolerances (as suggested by their present biogeographic distribution) were able to thrive under colder conditions increasing richness and evenness of LG nearshore assemblages (Figure 5). The subsequent Holocene climate warming reversed this pattern by again favoring Lusitanian and Mediterranean species, which dominated highly variable shallow‐marine environments in the Adriatic Sea during the last interglacial period. The species that were common in LG assemblages are still found in nearshore settings in northern Europe, but they likely retracted to slightly deeper habitats in the Mediterranean part of their range. Such bathymetric shifts are frequently documented among marine species in response to the ongoing SST rise and might constitute an important driver of community reorganization (e.g., Pinsky et al., 2013; Weinberg, 2005).

### 4.3 Conservation implications for the 21st century

Our results together with the paleoclimate data and climate change scenarios point to the potential adaptive capacities of the Adriatic nearshore mollusk communities to the limited near‐future global warming. During the last interglacial, SSTs in the Northern Atlantic (above 23.5°N latitude) were between 0.6 and 1.3 ± 0.5°C higher than during the pre‐industrial times (Hoffman et al., 2017). However, within the Mediterranean basin which is considered a climatic hotspot sensitive to radiative forcing which amplifies climatic trends, paleotemperature estimates point toward higher values. Alkenone‐derived SSTs for the late LIG in the central Adriatic were estimated at ~22°C (see Figure S1d), that is ~3.5°C higher than present‐day SSTs (i.e., 18.5°C, that is the mean value resulting from daily estimates obtained offshore southern Marche and northern Puglia regions from July 2011 to June 2015; see table 1 in Gizzi et al., 2016). In addition, the radiative forcing of greenhouse gasses below 4.5 W/m^2^, as predicted by representative concentration pathways (RCP) 2.6 and 4.5, should constrain near‐future, central Adriatic mean SST warming to less than 2°C (see Shaltout & Omstedt, 2014 for projected SST at the end of the 21st century in the Adriatic). Therefore, the resilience of targeted assemblages and strong similarities in many of the ecosystem features between the present and last interglacial, suggest that efforts aimed at limiting the radiative forcing of greenhouses gasses below 4.5 W/m^2^ (i.e., RCP 4.5 scenario), should result in a limited impact on the Adriatic nearshore deltaic mollusk communities. However, other anthropogenic stressors including bottom trawling (Eigaard et al., 2017; Pitcher et al., 2022), hypoxic events (Justić, 1991), coastal landscape modifications, and aquaculture (Slišković et al., 2021; Viero et al., 2019) have been affecting community composition of the Adriatic ecosystems since at least the mid‐20th century. These multifaceted stressors are shifting community composition far more strongly than natural environmental drivers did during the lastest Quaternary (e.g., Gallmetzer et al., 2019; Kowalewski et al., 2015; Lotze et al., 2011; Tomašových et al., 2020). The ongoing human restructuring of these ecosystems could push local assemblages beyond the historical range of variability despite their high resilience to natural climate dynamics.

The long‐term perspective offered by geohistorical archives is fundamental for defining ecological baselines, which in turn should inform conservation actions aimed at sustaining highly dynamic coastal ecosystems. However, restoration of environments and resource stocks to the pristine or pre‐industrial conditions may not be feasible given the socio‐economic contexts of these densely populated areas. Long‐term conservation practices, therefore, should focus on maintaining connectivity among areas of relatively unaffected, natural habitats that could act as a buffer against ecosystem shifts due to ongoing climate warming. Such low impact areas increase habitat heterogeneity across different climatic zones and can serve as potential thermal refugia, thus promoting resilience to climate change (Bernhardt & Leslie, 2013). Maintaining and possibly improving the quality of marine refugia in the Mediterranean Sea (Mu & Wilcove, 2020) is thus necessary to preserve the structure and resilience of coastal communities and their ecosystem services (Schneider, 2018).

In summary, this study suggests that the Adriatic nearshore assemblages have alternated naturally between two community states over the last ~125 kyr and thus demonstrated a remarkable resilience in face of major, long‐term environmental perturbations. The observed resilience during the most recent interglacial–glacial transitions is not consistent with stochastic or interaction‐based community assembly models. Instead, the high similarity between assemblages representing the two interglacial periods and distinct composition observed in the glacial faunas suggest that, over millennial timescales, shallow‐marine benthic assemblages have been primarily structured by environmental forcing. Over the last century, however, pollution, eutrophication, trawling, and invasive species have been affecting coastal ecosystems. Our findings suggest that if these impacts can be controlled, the targeted nearshore communities of the Adriatic should be resilient to the limited rise of sea surface temperatures predicted for the near future. In addition to the international policies addressing global warming, we stress here the importance of the mitigation of the threats associated with human activities in the coastal areas at the local and regional levels.

## CONFLICT OF INTEREST

The authors declare that there is no conflict of interest.

## AUTHOR CONTRIBUTIONS

DS, RN, and MK involved in conceptualization, methodology, and visualization of the study; DS, MA, RN, CP, and FG investigated the study; DS, RN, MK, and MA involved in writing—original draft; MK, RN, DS, CP, FG, and FT involved in writing—review & editing.

## Supporting information

Supplementary Material

## Data Availability

The data that support the findings of this study are available at 10.5061/dryad.mpg4f4r2c.

## References

[gcb16168-bib-0001] Amorosi, A. , Maselli, V. , & Trincardi, F. (2016). Onshore to offshore anatomy of a late Quaternary source‐to‐sink system (Po Plain‐Adriatic Sea, Italy). Earth‐Science Reviews, 153, 212–237. 10.1016/j.earscirev.2015.10.010

[gcb16168-bib-0002] Anderson, M. J. , & Walsh, D. C. (2013). PERMANOVA, ANOSIM, and the Mantel test in the face of heterogeneous dispersions: What null hypothesis are you testing? Ecological Monographs, 83(4), 557–574. 10.1890/12-2010.1

[gcb16168-bib-0003] Aronson, R. B. , & Precht, W. F. (2016). Physical and biological drivers of coral‐reef dynamics. In D. K. Hubbard , C. S. Rogers , J. H. Lipps , & G. D. Stanley (Eds.), Coral Reefs of the World (pp. 261–275). Springer.

[gcb16168-bib-0004] Asioli, A. , Trincardi, F. , Lowe, J. J. , Ariztegui, D. , Langone, L. , & Oldfield, F. (2001). Sub‐millennial scale climatic oscillations in the central Adriatic during the Lateglacial: Palaeoceanographic implications. Quaternary Science Review, 20, 1201–1221. 10.1016/S0277-3791(00)00147-5

[gcb16168-bib-0005] Barnosky, A. D. , Hadly, E. A. , Gonzalez, P. , Head, J. , Polly, P. D. , Lawing, A. M. , Eronen, J. T. , Ackerly, D. D. , Alex, K. , Biber, E. , Blois, J. , Brashares, J. , Ceballos, G. , Davis, E. , Dietl, G. P. , Dirzo, R. , Doremus, H. , Fortelius, M. , Greene, H. W. , … Zhang, Z. (2017). Merging paleobiology with conservation biology to guide the future of terrestrial ecosystems. Science, 355(6325), eaah4787. 10.1126/science.aah4787 28183912

[gcb16168-bib-0006] Bedulli, D. , Dell’Angelo, B. , Piani, P. , Spada, G. , Zurlini, G. , & Bruschi, A. (1984). Census of the distribution of the Italian Marine Mollusca. Nova Thalassia, suppl. 6, 585–590.

[gcb16168-bib-0007] Bernhardt, J. R. , & Leslie, H. M. (2013). Resilience to climate change in coastal marine ecosystems. Annual Review of Marine Science, 5, 371–392. 10.1146/annurev-marine-121211-172411 22809195

[gcb16168-bib-0008] Cannariato, K. G. , Kennett, J. P. , & Behl, R. J. (1999). Biotic response to late Quaternary rapid climate switches in Santa Barbara Basin: Ecological and evolutionary implications. Geology, 27(1), 63–66. 10.1130/0091-7613(1999)027<0063:BRTLQR>2.3.CO;2

[gcb16168-bib-0009] Capotondi, L. (2004). Marine sea surface paleotemperature. In F. Antonioli , & G. Vai (Ed.), Climex Maps explanatory notes (pp. 1–4). LAC Firenze.

[gcb16168-bib-0010] Danise, S. , & Holland, S. M. (2017). Faunal response to sea‐level and climate change in a short‐lived seaway: Jurassic of the Western Interior, USA. Palaeontology, 60(2), 213–232. 10.1111/pala.12278 28781385 PMC5518760

[gcb16168-bib-0011] Davies, A. L. , Streeter, R. , Lawson, I. T. , Roucoux, K. H. , & Hiles, W. (2018). The application of resilience concepts in palaeoecology. The Holocene, 28(9), 1523–1534. 10.1177/0959683618777077

[gcb16168-bib-0012] Dillon, E. M. , Lafferty, K. D. , McCauley, D. J. , Bradley, D. , Norris, R. D. , Caselle, J. E. , DiRenzo, G. V. , Gardner, J. P. A. , & O'Dea, A. (2020). Dermal denticle assemblages in coral reef sediments correlate with conventional shark surveys. Methods in Ecology and Evolution, 11(3), 362–375. 10.1111/2041-210X.13346

[gcb16168-bib-0013] Doney, S. C. , Ruckelshaus, M. , Emmett Duffy, J. , Barry, J. P. , Chan, F. , English, C. A. , Galindo, H. M. , Grebmeier, J. M. , Hollowed, A. B. , Knowlton, N. , Polovina, J. , Rabalais, N. N. , Sydeman, W. J. , & Talley, L. D. (2012). Climate change impacts on marine ecosystems. Annual Review of Marine Science, 4, 11–37. 10.1146/annurev-marine-041911-111611 22457967

[gcb16168-bib-0014] Eigaard, O. R. , Bastardie, F. , Hintzen, N. T. , Buhl‐Mortensen, L. , Buhl‐Mortensen, P. , Catarino, R. , Dinesen, G. E. , Egekvist, J. , Fock, H. O. , Geitner, K. , Gerritsen, H. D. , González, M. M. , Jonsson, P. , Kavadas, S. , Laffargue, P. , Lundy, M. , Gonzalez‐Mirelis, G. , Nielsen, J. R. , Papadopoulou, N. , … Rijnsdorp, A. D. (2017). The footprint of bottom trawling in European waters: Distribution, intensity, and seabed integrity. ICES Journal of Marine Science, 74(3), 847–865. 10.1093/icesjms/fsw194

[gcb16168-bib-0015] Fredston‐Hermann, A. , Gaines, S. D. , & Halpern, B. S. (2018). Biogeographic constraints to marine conservation in a changing climate. Annals of the New York Academy of Sciences, 1429(1), 5–17. 10.1111/nyas.13597 29411385

[gcb16168-bib-0016] Friedman, W. R. , Halpern, B. S. , McLeod, E. , Beck, M. W. , Duarte, C. M. , Kappel, C. V. , Levine, A. , Sluka, R. D. , Adler, S. , O’Hara, C. C. , Sterling, E. J. , Tapia‐Lewin, S. , Losada, I. J. , McClanahan, T. R. , Pendleton, L. , Spring, M. , Toomey, J. P. , Weiss, K. R. , Possingham, H. P. , & Montambault, J. R. (2020). Research priorities for achieving healthy marine ecosystems and human communities in a changing climate. Frontiers in Marine Science, 7, 1–5. 10.3389/fmars.2020.00005 32802822

[gcb16168-bib-0017] Gallmetzer, I. , Haselmair, A. , Tomašových, A. , Mautner, A.‐K. , Schnedl, S.‐M. , Cassin, D. , Zonta, R. , & Zuschin, M. (2019). Tracing origin and collapse of Holocene benthic baseline communities in the northern Adriatic Sea. Palaios, 34(3), 121–145. 10.2110/palo.2018.068

[gcb16168-bib-0018] Gizzi, F. , Caccia, M. G. , Simoncini, G. A. , Mancuso, A. , Reggi, M. , Fermani, S. , Brizi, L. , Fantazzini, P. , Stagioni, M. , Falini, G. , Piccinetti, C. , & Goffredo, S. (2016). Shell properties of commercial clam *Chamelea gallina* are influenced by temperature and solar radiation along a wide latitudinal gradient. Scientific Reports, 6(1), 1–12. 10.1038/srep36420 27805037 PMC5090357

[gcb16168-bib-0019] Graham, N. A. , Cinner, J. E. , Norström, A. V. , & Nyström, M. (2014). Coral reefs as novel ecosystems: Embracing new futures. Current Opinion in Environmental Sustainability, 7, 9–14. 10.1016/j.cosust.2013.11.023

[gcb16168-bib-0020] Griffith, G. P. , Strutton, P. G. , & Semmens, J. M. (2018). Climate change alters stability and species potential interactions in a large marine ecosystem. Global Change Biology, 24(1), e90–e100. 10.1111/gcb.13891 28869695

[gcb16168-bib-0021] Grime, J. P. , Fridley, J. D. , Askew, A. P. , Thompson, K. , Hodgson, J. G. , & Bennett, C. R. (2008). Long‐term resistance to simulated climate change in an infertile grassland. Proceedings of the National Academy of Sciences of the United States of America, 105(29), 10028–10032. 10.1073pnas.0711567105 18606995 10.1073/pnas.0711567105 PMC2481365

[gcb16168-bib-0022] Grimm, V. , & Wissel, C. (1997). Babel, or the ecological stability discussions: an inventory and analysis of terminology and a guide for avoiding confusion. Oecologia, 109(3), 323–334. 10.1007/s004420050090 28307528

[gcb16168-bib-0023] Harnik, P. G. , Lotze, H. K. , Anderson, S. C. , Finkel, Z. V. , Finnegan, S. , Lindberg, D. R. , Liow, L. H. , Lockwood, R. , McClain, C. R. , McGuire, J. L. , O’Dea, A. , Pandolfi, J. M. , Simpson, C. , & Tittensor, D. P. (2012). Extinctions in ancient and modern seas. Trends in Ecology & Evolution, 27(11), 608–617. 10.1016/j.tree.2012.07.010 22889500

[gcb16168-bib-0024] Hoffman, J. S. , Clark, P. U. , Parnell, A. C. , & He, F. (2017). Regional and global sea‐surface temperatures during the last interglaciation. Science, 355(6322), 276–279. 10.1126/science.aai8464 28104887

[gcb16168-bib-0025] Hyman, A. C. , Frazer, T. K. , Jacoby, C. A. , Frost, J. R. , & Kowalewski, M. (2019). Long‐term persistence of structured habitats: seagrass meadows as enduring hotspots of biodiversity and faunal stability. Proceedings of the Royal Society B, 286(1912), 20191861. 10.1098/rspb.2019.1861 31575365 PMC6790775

[gcb16168-bib-0026] Jackson, S. T. , & Blois, J. L. (2015). Community ecology in a changing environment: Perspectives from the Quaternary. Proceedings of the National Academy of Sciences of the United States of America, 112(16), 4915–4921. 10.1073/pnas.1403664111 25901314 PMC4413336

[gcb16168-bib-0027] Justić, D. (1991). Hypoxic conditions in the northern Adriatic Sea: historical development and ecological significance. Geological Society, London, Special Publications, 58, 95–105. 10.1144/GSL.SP.1991.058.01.07

[gcb16168-bib-0028] Kidwell, S. M. (2015 ). Biology in the Anthropocene: Challenges and insights from young fossil records. Proceedings of the National Academy of Sciences of the United States of America, 112(16), 4922–4929. 10.1073/pnas.1403660112 25901315 PMC4413286

[gcb16168-bib-0029] Kitamura, A. , Omote, H. , & Oda, M. (2000). Molluscan response to early Pleistocene rapid warming in the Sea of Japan. Geology, 28(8), 723–726. 10.1130/0091-7613(2000)28<723:MRTEPR>2.0.CO;2

[gcb16168-bib-0030] Kowalewski, M. , Wittmer, J. M. , Dexter, T. A. , Amorosi, A. , & Scarponi, D. (2015). Differential responses of marine communities to natural and anthropogenic changes. Proceedings of the Royal Society B: Biological Sciences, 282(1803), 20142990. 10.1098/rspb.2014.2990 PMC4345463 25673689

[gcb16168-bib-0031] Lotze, H. K. , Coll, M. , & Dunne, J. A. (2011). Historical changes in marine resources, food‐web structure and ecosystem functioning in the Adriatic Sea, Mediterranean. Ecosystems, 14(2), 198–222. 10.1007/s10021-010-9404-8

[gcb16168-bib-0032] Martinelli, J. C. , Soto, L. P. , González, J. , & Rivadeneira, M. M. (2017). Benthic communities under anthropogenic pressure show resilience across the Quaternary. Royal Society Open Science, 4(9), 170796. 10.1098/rsos.170796. 28989781 PMC5627121

[gcb16168-bib-0033] Maselli, V. , Trincardi, F. , Asioli, A. , Ceregato, A. , Rizzetto, F. , & Taviani, M. (2014). Delta growth and river valleys: The influence of climate and sea level changes on the South Adriatic shelf (Mediterranean Sea). Quaternary Science Reviews, 99, 146–163. 10.1016/j.quascirev.2014.06.014

[gcb16168-bib-0034] McKinney, F. K. (2007). The northern Adriatic ecosystem: Deep time in a shallow sea. Columbia University Press, 9780231132428.

[gcb16168-bib-0035] Monegatti, P. , & Raffi, S. (2001). Taxonomic diversity and stratigraphic distribution of Mediterranean Pliocene bivalves. Palaeogeography, Palaeoclimatology, Palaeoecology, 165, 171–193. 10.1016/S0031-0182(00)00159-0

[gcb16168-bib-0036] Mu, T. , & Wilcove, D. S. (2020). Upper tidal flats are disproportionately important for the conservation of migratory shorebirds. Proceedings of the Royal Society B, 287(1928), 20200278. 10.1098/rspb.2020.0278rspb20200278 32486983 PMC7341910

[gcb16168-bib-0037] Nawrot, R. , Scarponi, D. , Azzarone, M. , Dexter, T. A. , Kusnerik, K. M. , Wittmer, J. M. , Amorosi, A. , & Kowalewski, M. (2018). Stratigraphic signatures of mass extinctions: ecological and sedimentary determinants. Proceedings of the Royal Society B: Biological Sciences, 285(1886), 20181191. 10.1098/rspb.2018.1191 PMC6158527 30209225

[gcb16168-bib-0038] Nikanorov, A. M. , & Sukhorukov, B. L. (2008). Ecological hysteresis. Doklady Earth Sciences, 423(1), 1–1282. 10.1134/S1028334X08080229

[gcb16168-bib-0039] Oksanen, J. , Blanchet, F. G. , Friendly, M. , Kindt, R. , Legendre, P. , McGlinn, D. , Minchin, P. R. , O'Hara, R. B. , Simpson, G. L. , Solymos, P. , Henry, M. , Stevens, H. , Szoecs, E. , & Wagner, H. (2018). Vegan: Community Ecology Package. R package version 2.5‐6. https://CRAN.R-project.org/package=vegan

[gcb16168-bib-0040] O'Leary, J. K. , Micheli, F. , Airoldi, L. , Boch, C. , De Leo, G. , Elahi, R. , Ferretti, F. , Graham, N. A. J. , Litvin, S. Y. , Low, N. H. , Lummis, S. , Nickols, K. J. , & Wong, J. (2017). The resilience of marine ecosystems to climatic disturbances. BioScience, 67(3), 208–220. 10.1093/biosci/biw161

[gcb16168-bib-0041] Pandolfi, J. M. (1996). Limited membership in Pleistocene reef coral assemblages from the Huon Peninsula, Papua New Guinea: Constancy during global change. Paleobiology, 22(2), 152–176. 10.1017/S0094837300016158

[gcb16168-bib-0042] Pellegrini, C. , Asioli, A. , Bohacs, K. M. , Drexler, T. M. , Howard, R. F. , Sweet, M. L. , Maselli, V. , Rovere, M. , Gamberi, F. , Dalla Valle, G. , & Trincardi, F. (2018). The late Pleistocene Po River lowstand wedge in the Adriatic Sea: Controls on architecture variability and sediment partitioning. Marine and Petroleum Geology, 96, 16–50. 10.1016/j.marpetgeo.2018.03.002

[gcb16168-bib-0043] Pellegrini, C. , Maselli, V. , Gamberi, F. , Asioli, A. , Bohacs, K. M. , Drexler, T. M. , & Trincardi, F. (2017). How to make a 350‐m‐thick lowstand systems tract in 17,000 years: The Late Pleistocene Po River (Italy) lowstand wedge. Geology, 45(4), 327–330. 10.1130/G38848.1

[gcb16168-bib-0044] Pellegrini, C. , Tesi, T. , Schieber, J. , Bohacs, K. M. , Rovere, M. , Asioli, A. , Nogarotto, A. , & Trincardi, F. (2021). Fate of terrigenous organic carbon in muddy clinothems on continental shelves revealed by stratal geometries: Insight from the Adriatic sedimentary archive. Global and Planetary Change, 203, 103539. 10.1016/j.gloplacha.2021.103539.

[gcb16168-bib-0045] Pérès, J. M. , & Picard, J. (1964). Nouveau manuel de bionomie benthique de la mer Méditerranée. Recueil des Travaux de la Station marine d'Endoume, 31(47), 5–137.

[gcb16168-bib-0046] Petersen, C. G. (1913). The animal communities of the sea bottom and their importance for marine zoogeography. Report from the Danish Biological Station, 21, 1–68.

[gcb16168-bib-0047] Pinsky, M. L. , Worm, B. , Fogarty, J. F. , Sarmiento, J. L. , & Levin, S. A. (2013). Taxa track local climate velocities. Science, 241(6151), 1239–1242. 10.1126/science.1239352 24031017

[gcb16168-bib-0048] Pitcher, C. R. , Hiddink, J. G. , Jennings, S. , Collie, J. , Parma, A. M. , Amoroso, R. , Mazor, T. , Sciberras, M. , McConnaughey, R. A. , Rijnsdorp, A. D. , Kaiser, M. J. , Suuronen, P. , & Hilborn, R. (2022). Trawl impacts on the relative status of biotic communities of seabed sedimentary habitats in 24 regions worldwide. Proceedings of the National Academy of Sciences, 119(2), e2109449119. 10.1073/pnas.2109449119 PMC8764683 34983873

[gcb16168-bib-0049] Piva, A. , Asioli, A. , Schneider, R. R. , Trincardi, F. , Andersen, N. , Colmenero‐Hidalgo, E. , & Vigliotti, L. (2008). Climatic cycles as expressed in sediments of the PROMESS1 borehole PRAD1‐2, central Adriatic, for the last 370 ka: 1. Integrated stratigraphy. Geochemistry, Geophysics, Geosystems, 9(1), Q01R01. 10.1029/2007GC001785

[gcb16168-bib-0050] Poppe, G. T. , & Goto, Y. (1991). European seashells, vol. I. Verlag Christa Hemmen.

[gcb16168-bib-0051] Poppe, G. T. , & Goto, Y. (1993). European seashells, vol. II. ConchBooks.

[gcb16168-bib-0052] R Development Team (2018). R: A language and environment for statistical computing. R Foundation for Statistical Computing. https://www.R‐project.org/

[gcb16168-bib-0053] Rakocinski, C. , Heard, R. W. , Simons, T. , & Gledhill, D. (1991). Macroinvertebrate associations from beaches of selected barrier islands in the northern Gulf of Mexico: important environmental relationships. Bulletin of Marine Science, 48(3), 689–701.

[gcb16168-bib-0054] Rocca, J. D. , Simonin, M. , Bernhardt, E. S. , Washburne, A. D. , & Wright, J. P. (2020). Rare microbial taxa emerge when communities collide: freshwater and marine microbiome responses to experimental mixing. Ecology, 101(3), e02956. 10.1002/ecy.2956 31840237

[gcb16168-bib-0055] Schneider, C. L. (2018). Marine refugia past, present, and future: Lessons from ancient geologic crises for modern marine ecosystem conservation. In C. Tyler , & C. Schneider (Eds.), Marine Conservation Paleobiology (pp. 163–208), Springer, ISBN: 978‐3‐319‐73795‐9

[gcb16168-bib-0056] Shaltout, M. , & Omstedt, A. (2014). Recent sea surface temperature trends and future scenarios for the Mediterranean Sea. Oceanologia, 56(3), 411–443. 10.5697/oc.56-3.411

[gcb16168-bib-0057] Slišković, M. , Piria, M. , Nerlović, V. , Ivelja, K. P. , Gavrilović, A. , & Mrčelić, G. J. (2021). Non‐indigenous species likely introduced by shipping into the Adriatic Sea. Marine Policy, 129, 104516. 10.1016/j.marpol.2021.104516

[gcb16168-bib-0058] Stanton, R. J. , & Dodd, J. R. (1997). Lack of stasis in late Cenozoic marine faunas and communities, central California. Lethaia, 30(3), 239–256. 10.1111/j.1502-3931.1997.tb00466.x

[gcb16168-bib-0059] Steger, J. , Bošnjak, M. , Belmaker, J. , Galil, B. S. , Zuschin, M. , Albano, P. G. , & Soininen, J. (2022). Non‐indigenous molluscs in the Eastern Mediterranean have distinct traits and cannot replace historic ecosystem functioning. Global Ecology and Biogeography, 31(1), 89–102. 10.1111/geb.13415

[gcb16168-bib-0060] Tager, D. , Webster, J. M. , Potts, D. C. , Renema, W. , Braga, J. C. , & Pandolfi, J. M. (2010). Community dynamics of Pleistocene coral reefs during alternative climatic regimes. Ecology, 91(1), 191–200. 10.1890/08-0422.1 20380208

[gcb16168-bib-0061] Tomašových, A. , Albano, P. G. , Fuksi, T. , Gallmetzer, I. , Haselmair, A. , Kowalewski, M. , Nawrot, R. , Nerlović, V. , Scarponi, D. , & Zuschin, M. (2020). Ecological regime shift preserved in the Anthropocene stratigraphic record. Proceedings of the Royal Society B, 287(1929), 20200695. 10.1098/rspb.2020.0695rspb20200695 32546093 PMC7329033

[gcb16168-bib-0062] Trisos, C. H. , Merow, C. , & Pigot, A. L. (2020). The projected timing of abrupt ecological disruption from climate change. Nature, 580(7804), 496–501. 10.1038/s41586-020-2189-9 32322063

[gcb16168-bib-0063] Vellend, M. (2016). The theory of ecological communities (MPB‐57). Princeton University Press, ISBN 1400883792, 9781400883790.

[gcb16168-bib-0064] Viero, D. P. , Roder, G. , Matticchio, B. , Defina, A. , & Tarolli, P. (2019). Floods, landscape modifications and population dynamics in anthropogenic coastal lowlands: The Polesine (northern Italy) case study. Science of the Total Environment, 651, 1435–1450. 10.1016/j.scitotenv.2018.09.121 30360273

[gcb16168-bib-0065] Weinberg, J. R. (2005). Bathymetric shift in the distribution of Atlantic surfclams: response to warmer ocean temperature. ICES Journal of Marine Science, 62, 1444e1453. 10.1016/j.icesjms.2005.04.020

[gcb16168-bib-0066] Wittmer, J. M. , Dexter, T. A. , Scarponi, D. , Amorosi, A. , & Kowalewski, M. (2014). Quantitative bathymetric models for late Quaternary transgressive‐regressive cycles of the Po Plain, Italy. The Journal of Geology, 122(6), 649–670. 10.1086/677901

