# Supplementary material for: Resilient biotic response to long‐term climate change in the Adriatic Sea
Source: Glob Chang Biol. 2022 Apr 12;28(13):4041–53. doi: 10.1111/gcb.16168 (PMC9324144; doi:10.1111/gcb.16168)
Supplement: Supplementary file 1 — Supplementary Material [file GCB-28-4041-s001.docx]

Supplementary materials for

**Resilient biotic response to long-term climate change in the Adriatic Sea**

Daniele Scarponi, * Rafał Nawrot, Michele Azzarone, Claudio Pellegrini, Fabiano Gamberi, Fabio Trincardi, Michał Kowalewski

*Corresponding author. Email: daniele.scarponi@unibo.it

**This PDF file includes:**

Supplementary Text S1-S2

Figs. S1 to S6

Tables S1 to S6

Appendixes S1-S2 (i.e., datasets saved as .csv files), and R script files, are downloadable here:

<https://datadryad.org/stash/share/Q4S-1eQTV0D0JYSvpbme6VRTfoS9QXahvcR-dV4rGJ4>

**Supplementary Text**

**S1-Study Area. The Adriatic coastal system since the last interglacial**

The Adriatic is an elongated (~800 km) and narrow semi-enclosed epicontinental basin (Fig. S1). The northern sector of the basin is characterised by a wide shelf with a low 0.02° topographic gradient and extends southward for ~350 km from the Gulf of Venice. The central sector of the basin, which is confined to the south by the Gallignani-Pelagosa ridge, displays a narrower shelf (< 50 km), with steeper gradient (<0.5°), that border two small remnant basins, the Mid Adriatic Deep (MAD) and southern Adriatic Deep (SAD). The former was partially filled by the Po River when, at times of sea-level lowstand, its Delta was repeatedly located at the edge of the MAD (Ridente et al., 2007). The southern Adriatic sector (SAD), south of Pelagosa Sill has a maximum depth of 1200 m. In this area, the shelf is very steep and narrow (<20 km) with irregular topography characterised by tectonic structures such as the Gondola Fault/Gondola anticline, the Dauno seamount and the Bari Canyon; the latter is the main sediment conduit on the southwestern side of the Adriatic Sea (e.g., Pellegrini et al., 2016). In the southernmost part of the Adriatic, the Otranto Strait enables connection with the Mediterranean Sea. As reported by Artegiani et al. (1997), this narrow strait plays a pivotal role in controlling the modern-day circulation pattern of not only the Adriatic but of the entire Mediterranean Sea. The western side of the Adriatic basin and its sedimentary dynamics are mainly influenced by the Po river, that is the longest river in Italy and flows eastward into the northern Adriatic sea. The Po river-Adriatic sea system forms a sedimentary tract, very sensitive to glacial interglacial variations which shaped the continental margin of the Adriatic during the late Quaternary.

*The Po River-Adriatic Sea system since the last interglacial*

The modern Po Delta (Fig. S1), with a surface of 54,000 ha, forms the largest complex of wetlands in Italy and is localized at the border between the Emilia-Romagna and Veneto regions. The Po Delta includes an extensive delta plain, a river-influenced delta front, and a broad asymmetric composite prodelta. The sediment dispersal is controlled by six main distributary channels which form an equal number of main prodelta lobes. The modern Po Delta is a relatively recent geomorphologic element that evolved during the last ~900 years, mainly in response to the Porto Viro diversion (1600-1604 AD; Correggiari et al., 2005). South of the Po Delta, the coastal plain shows a triangular shape, tapering southward, as the Apennine chain meets the coastline. During the Middle-Late Holocene, the location of the Po Delta main distributary channels was few dozens of kilometers south from their present-day position, north of Ravenna (Amorosi et al., 2003; 2017 and references therein). Similar palaeogeographic setting characterised the last interglacial phase, as evidenced by a several meter thick (2 to 10 m) and laterally extensive (>100 km) delta front and strandplain deposits recovered in the subsurface of the Po coastal plain just few kilometers south of the present-day location of the Po Delta (Campo et al., 2020 and references therein). The main sedimentary units of interest for this study are the Holocene and last interglacial deltaic and coastal successions (nearshore deposits, Table S2) located below the present-day Po coastal plain surface, south of the modern Po Delta (Fig. S1), and the late glacial deltaic and coastal succession preserved along the northern- and western-edges of the Adriatic shelf, at present situated below the Adriatic Sea at ca. 100-150 m below the sea-level (Fig. S1). Although the two study areas (Po coastal plain and Adriatic shelf margin) share a common geological and sedimentological evolution, they are treated separately since the main phases of marine sediment accumulation occur in them at different times. The Po coastal plain consists of wedge-shaped sedimentary units that were deposited mainly during sea-level highstands (Amorosi et al., 2020 and references therein). In contrast, the expanded deltaic and coastal successions of the Central and Southern Adriatic shelf margin were stacked during the last sea-level lowstand and the early phase of post-glacial transgression (Table S2). Hence they are characterised by short intervals of rapid sedimentation (e.g., ~3 m/kyr, net sedimentation rates obtained from topmost 2 meters of core LSD-2-26), linked to phases of sea-level fall and still-stand (i.e., Bølling-Allerød/Younger Dryas transition).

*Late Quaternary stratigraphic architecture of the Po coastal plain*

Within the latest Quaternary succession (<150 kyr) of the Po coastal plain, two wedge-shaped shallow marine sedimentary bodies assigned to the marine isotope stage (MIS) 5 and MIS 1 are encountered at depths of 0-30 m and 90-120 m below the surface. These bodies, corresponding respectively to the current (CIG) and last interglacial (LIG), show comparable transgressive-regressive stacking patterns and were deposited during the two major transgressive pulses and subsequent sea-level highstands of the last 150 kyr (Campo et al., 2020 and references therein). The two wedge-shaped units are separated by a 50-100 m thick succession of alluvial and fluvial sediments recording the overall sea-level fall and lowstand that occurred between ~116-~14 kyr, and resulted in extensive basinward shifts of alluvial facies. During the last glacial maximum (24-18 kyr), the Po Delta system and the Adriatic shoreline were adjacent to the MAD (Pellegrini et al., 2018 and references therein; Fig. S1) and the present-day Po coastal plain recorded a succession of laterally discontinuous fluvial sand bodies associated to pedogenically altered and extensive floodplain deposits (Amorosi et al., 2017). Within these wedge-shaped units, the transgressive surface, being associated with a series of palaeosols formed during the last phases of the cold period, is a regional stratigraphic surface which can be recognized in cores by means of lithologic and palaeobiologic insights (Amorosi et al., 2004). While the internal organization of the MIS-5e is not studied in detail due to the limited number of deeper cores available, the CIG succession was investigated in detail during the last decades thanks to a wealth of core and geophysical data (e.g., Scarponi & Kowalewski, 2004; Calabrese & Cibin, 2014; Campo et al., 2017; Greggio et al., 2017). A series of lagoon-barrier systems, a few meter thick, recording the post-glacial eustatic sea-level rise and the early phase of slow progradation of bay-head deltas within the back barrier areas, is overlain by an aggradational-to-increasingly-progradational succession of mainly coarse-grained coastal and deltaic facies, which represent the main target of this study. Hence, during the Middle Holocene the physiography of the study area transitioned from a barrier-lagoon-estuary system to wave-dominated coastal and deltaic systems (Bruno et al., 2017). These environmental dynamics are recorded in the sedimentary succession by the aggradational to slightly progradational stacking of beach ridges and delta front deposits, characterised by shallowing upward trends, passing inland to floodplain and wetland deposits. From 2.0 kyr cal BP till mid-20th century, the deltaic and coastal systems of Emilia-Romagna experienced a period of increased river discharge that promoted a strong progradation phase and the transition from wave-dominated to river-dominated deltaic systems shifting spatially due to multiple episodes of river avulsion (Amorosi et al., 2019).

*Late Quaternary stratigraphic architecture of the central and southern Adriatic shelf margin*

A network of high-resolution Uniboom 3.5-khz and Chirp-sonar profiles combined with lithostratigraphic and chronologically data from multiple short cores (Fig. S1; Table S1), forms the basis for detailed reconstruction of the Central Adriatic and MAD stratigraphic framework during the late Quaternary (Gamberi et al., 2020 and references therein). Similar to the Po coastal plain succession, the Middle Adriatic shelf edge records a cyclic staking pattern with alternating alluvial to coastal and open marine deposits that accumulated during glacial and interglacial periods, respectively. In the main study area, during the last glacial period, the largest amount of sediments was transported by the Po Delta main distributary channels and built the Po River Lowstand Wedge (PRLW), a ~350-m-thick succession that prograded 40 km southward in ~17 kyr (Fig. S1; Pellegrini et al., 2018 and references therein). The southward progradation of the PRLW was accompanied by the contemporary construction of minor sedimentary wedges on the edge of narrow shelves surrounding the flanks of the MAD and SAD, including that south of the Gargano promontory, which was sampled for this study (Fig. S1; Maselli et al., 2014). These minor wedges were fed by rivers of the central and southern Apennine chain. This combined buildup of sedimentary wedges severely reduced the MAD extension and its depth from ~450 m to the modern-day ~260 m (Pellegrini et al., 2018). During the last lowstand the central and southern Adriatic basins remained connected to the Mediterranean, thereby maintaining a link to global sea level, as recorded by the shifts in shoreline position and depositional environments. The PRLW, which represents the site of the majority of the samples for this study, is delimited at the base by the sequence boundary dated at ~31 kyr cal BP and at the top by the transgressive surface dated ~14.5 kyr cal BP (Pellegrini et al., 2018). The internal stratal architecture of the PRLW is composed of 13 elemental clinothems (from A1 to C2) displaying an overall progradational-to-aggradational stacking pattern. The details of clinothem features and arrangement within PRLW are given in Pellegrini et al. (2018) and references therein. Based on the geometry and internal features the PRLW clinothems were grouped into two sets. The lower one is composed mainly by alternation of the clinothem types A and B. These units indicate sustained progradation (and moderate aggradation) with a flat shelf-edge trajectory that led to progressive filling of the MAD depocenter. Conversely, the upper clinothem set, the target of this study, is represented by type C units that records limited progradation and strong aggradation as evidenced by the strong ascending trajectory of the shelf-edge that developed between 18 and ~14.5 kyr cal BP during the initial phase of sea-level rise following the Last Glacial Maximum (Azzarone et al., 2020). The post-Last Glacial Maximum (LGM) eustatic rise, with rates of up to 12 m/ky (Maselli et al., 2017), led to the abandonment of the PRLW close to the onset of Termination I after ~14.4 kyr cal BP. The core samples from SAD and analysed in this study (cores INV12 and MAP12 in Table S2) represent deposits of an asymmetric delta, currently located at mid-outer shelf depths south of the Gargano promontory (Maselli et al., 2014). The delta formed during the last glacial and supplied sandy material to the shelf until the Early Holocene when sea level rise reached the inner shelf, drowning the incised valley and forming a sheltered mini basin, which was subsequently filled by coalescing bayhead deltas (Maselli et al., 2014).

**S2 R-scripts and datasets (.csv files)**

**Appendix S1** Raw abundance data and stratigraphic information concerning investigated nearshore samples from the latest Quaternary sedimentary succession of the Adriatic basin. The appendix is downloadable as .csv file https://datadryad.org/stash/share/Q4S-1eQTV0D0JYSvpbme6VRTfoS9QXahvcR-dV4rGJ4 .Abbreviations: ID―taxa identification number also reported in the first row of the Potaxa; Region categorization: EMR―Emilia-Romagna, VT―Veneto, PU―Puglia, MAR―Marche, AB―Abruzzo; Systems tract: HST―Highstand systems tract, LST―Lowstand systems tract, e-TST―early Transgressive systems tract.

**Appendix S2** Ecologic and taxonomic information concerning all taxa of the Po-Adriatic dataset (i.e., 787). Please note that the study targeted nearshore samples, so only a restricted taxonomic pool of the 787 taxa shows abundance values. Within each Class, taxa are in alphabetical order (family level). Explanation of the acronyms is reported on the second sheet of the excel file. The appendix is downloadable as .csv and xlsx files <https://datadryad.org/stash/share/PzlOJnHlAuwAGDLwQ3M_Q76SDi9QUgWYB59d8FuT1po>

Abbreviations, Ecosystem categorization: M―marine, B―brackish, F―freshwater, L―land; Taxonomy categorization (Class/Phylum): P―Polyplachophora, G―Gastropoda, C―Cephalopoda, B―Bivalvia, S―Scaphopoda, Po―Polychaeta, Ma―Maxillpoda, Br―Brachiopoda, Ec―Echinoidea; Substrate categorization: IN―infaunal, SI―semi-infaunal, EP―epifaunal, WB―borer/nestler, EP-IN―epifaunal and infaunal behaviour, NC―nektonic; Mobility categorization: IM―immobile, SE―sedentary, AM―Actively mobile; Fixation categorization: UN―unattached, BA―byssaly attached, CE―cemented; Feeding categorisation: SU―suspension feeder, DE―(sub)surface (chemosymbiotic) deposit feeder, HE―herbivores, CAR―carnivores, OM―omnivorous, PAR―parasites, SU-DE―suspension and (sub)surface (chemosymbiotic) deposit feeder, PAR-CAR―parasites and carnivores.

**R.scripts** can be downloaded at: <https://datadryad.org/stash/share/PzlOJnHlAuwAGDLwQ3M_Q76SDi9QUgWYB59d8FuT1po>). Please download the two .csv files (i.e., Podata1 and Potaxa), and the four R-scripts in your R-working directory. Open with RStudio the file “Simple ecosystem models for fig 1” and run the R-code to obtain Figure 1 in pdf. Then open and run Po Resilience 2022 with RStudio to get the remaining figures and tables of the manuscript.

**SI Figures**


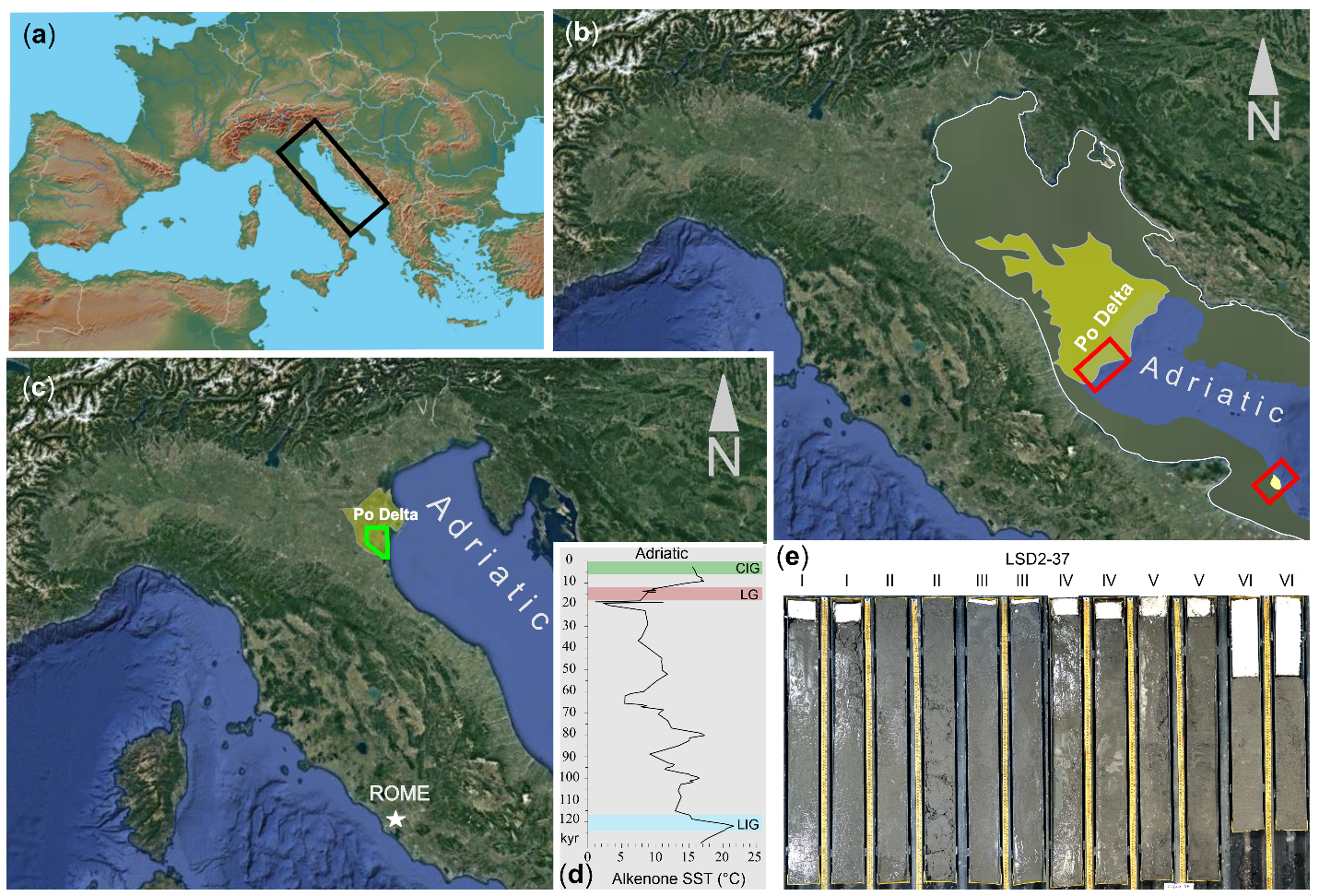


**Fig. S1.**

Study area with location (black rectangle) of the Adriatic Sea, (**a**). Targeted deltaic/coastal systems (in yellow) of maximum marine regression of the last glacial period (**b**) and of the present and last interglacial (**c**). Red rectangles (in **b**) show the location of the cored nearshore deposits of the last late glacial (<18 kyr cal BP). Green polygon (in **c**) shows the location of the cored nearshore deposits of Holocene and late Pleistocene age (i.e., current, and last interglacial). In the lower right corner: (**d**) alkenone-based trend in sea surface temperatures (SST) for the central Adriatic during the last 130 kyr and (**e**) a photo of one of the cores intersecting the uppermost Po river Lowstand Wedge of the Mid Adriatic Deep; each core segment (I-to-VI) has a length of 1 m. Abbreviation: SST=sea surface temperature.

**
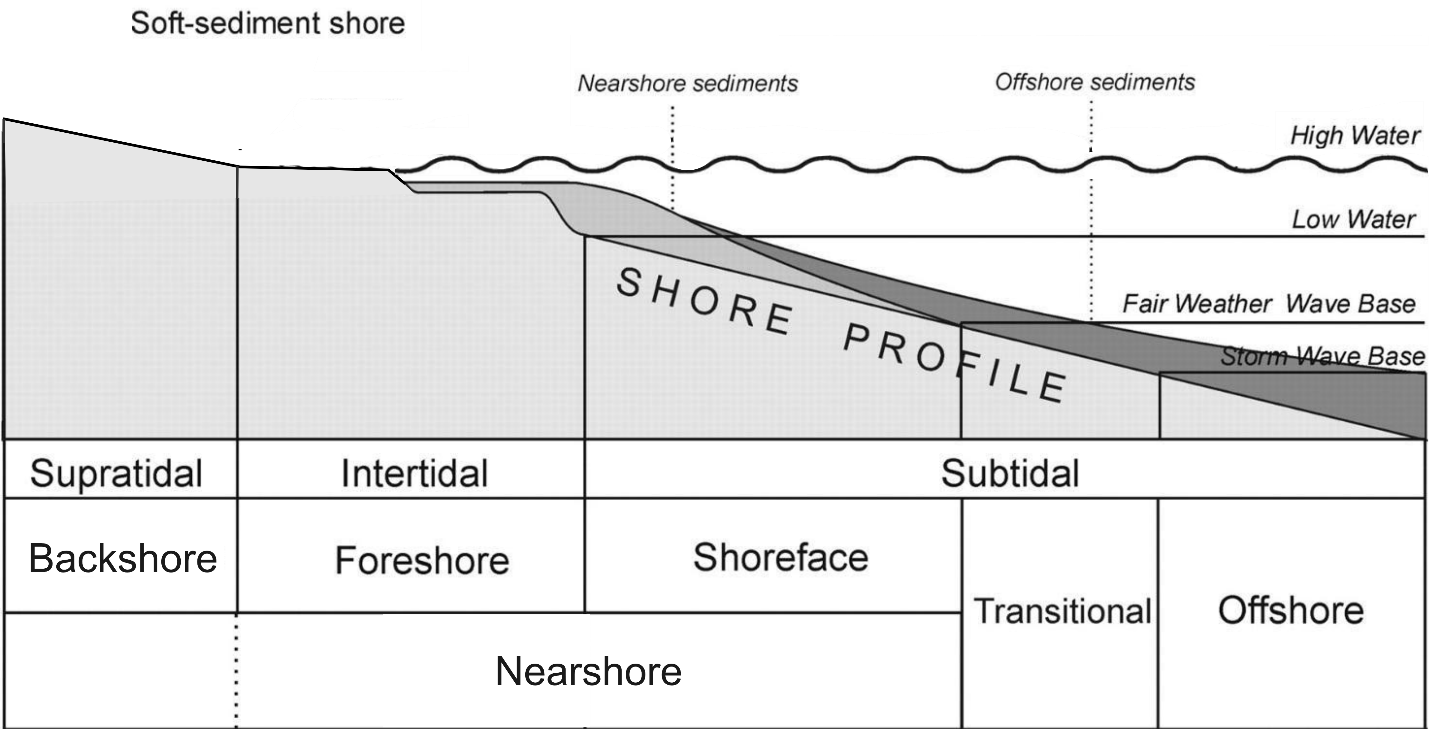
**

**Fig. S2.**

Nearshore settings. A collection of depositional environments found along an idealised transect from land to open shelf (modified after Sheppard, 2006). The studied samples represent soft-sediment nearshore environments, i.e., they comprise silty to coarse sandy deposits from variably fluvial influenced (deltaic) foreshore and shoreface settings.

**
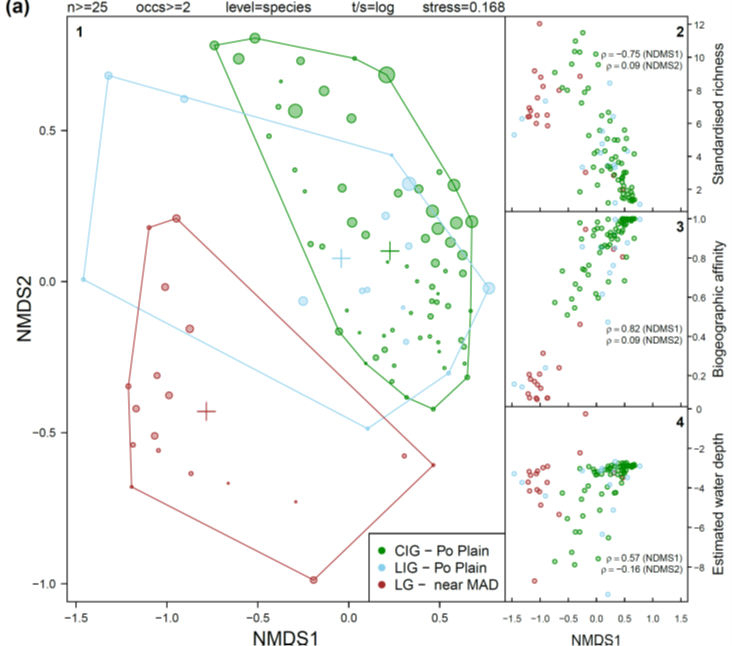
**

**
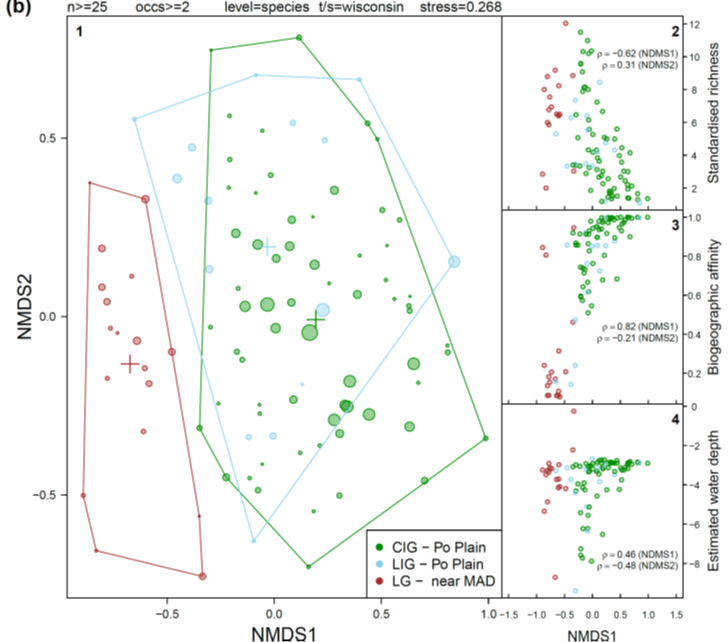
**

**Fig. S3.**

Non-metric Multidimensional Scaling outputs derived using Wisconsin double standardisation (panel **a**) and logarithm transformation (panel **b**) of the dataset, considering samples containing at least 25 specimens and species present in at least two samples. Samples are colour-coded according to the climatic interval: green―current interglacial (CIG), Light blue―last interglacial (LIG), and Dark red―late glacial (LG). In the NMDS output (1) the size of each point is proportional to sample size. Convex hulls delimit the ordination space occupied by each group of samples. 2) Correlation between NMDS axis 1 sample scores (NMDS1) and species richness rarefied to 25 specimens. Standardised species richness for relatively small samples tends to be primarily driven by evenness, so the two measures are strongly correlated. 3) Correlation between NMDS1 and relative abundance of Mediterranean-to-Lusitanian and West African species recovered in each sample. 4) Correlation between NMDS1 and the estimated water depth of the samples based on species bathymetric preferences (see Material and Methods for details). In 2, 3 and 4, rank correlation coefficient ρ is shown also for NMDS axis 2 sample scores.

**
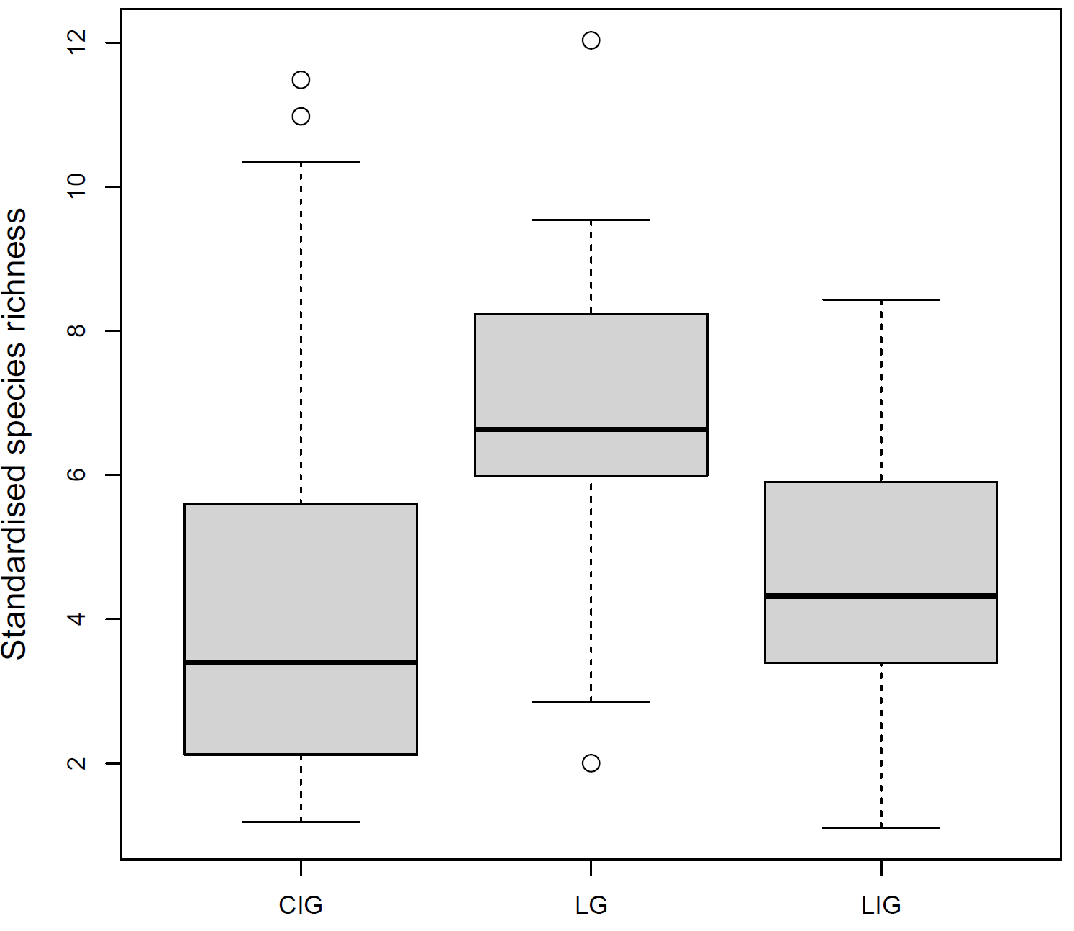
**

**Fig. S4.**

Sample-standardised richness estimates (n≥25 specimens) within nearshore setting at the three key-time intervals: LIG = last interglacial (Late Pleistocene); LG = last late glacial (Late Pleistocene); CIG = current interglacial (Holocene).

**
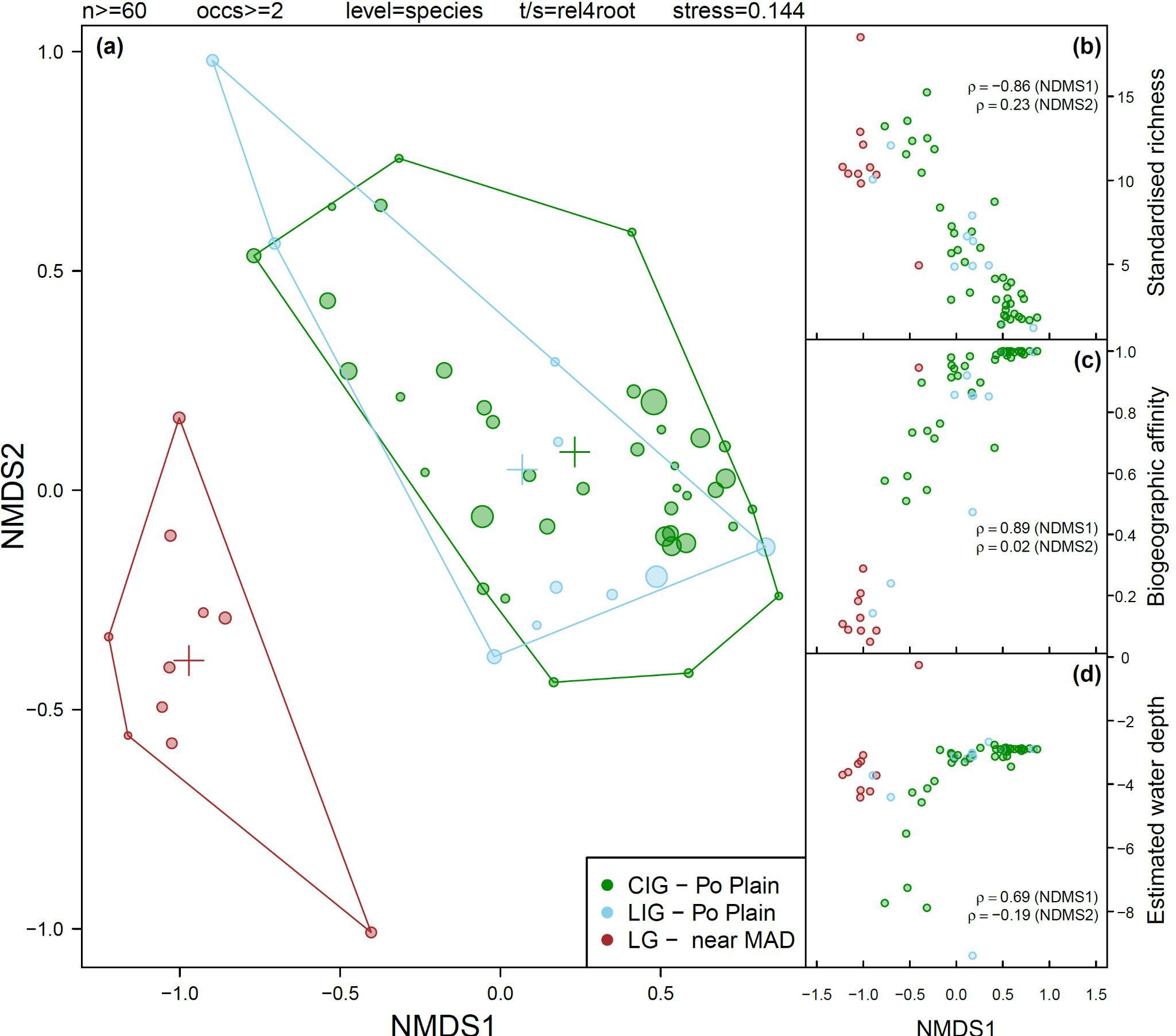
**

**Fig. S5.**

Non-metric Multidimensional Scaling – using higher sample threshold n of 60 specimens: (**a**) NMDS ordination based on the 4^th^-root transformed relative abundances of species (sample size ≥60 specimens). Symbols (solid circles) show samples from nearshore deposits representing the three time intervals: green―Holocene interglacial (CIG), light blue―Late Pleistocene interglacial (LIG), and dark red―Pleistocene last late glacial (LG). Convex hulls delimit the portion of the ordination space occupied by each group of samples. The size of each point depicts sample size. Convex hulls delimit the ordination space occupied by each group of samples; (**b**) Correlation between NMDS1 and species richness rarefied to 60 specimens; (**c**) Correlation between NMDS axis 1 sample scores (NMDS1) and relative abundance of Mediterranean-to-Lusitanian and West African species recovered in each sample; (**d**) Correlation between NMDS1 and the estimates of sample water depths based on species bathymetric preferences (see Methods for details). In **b**-**c** panels, rank correlation coefficient ρ is shown also for NMDS axis 2 sample scores.


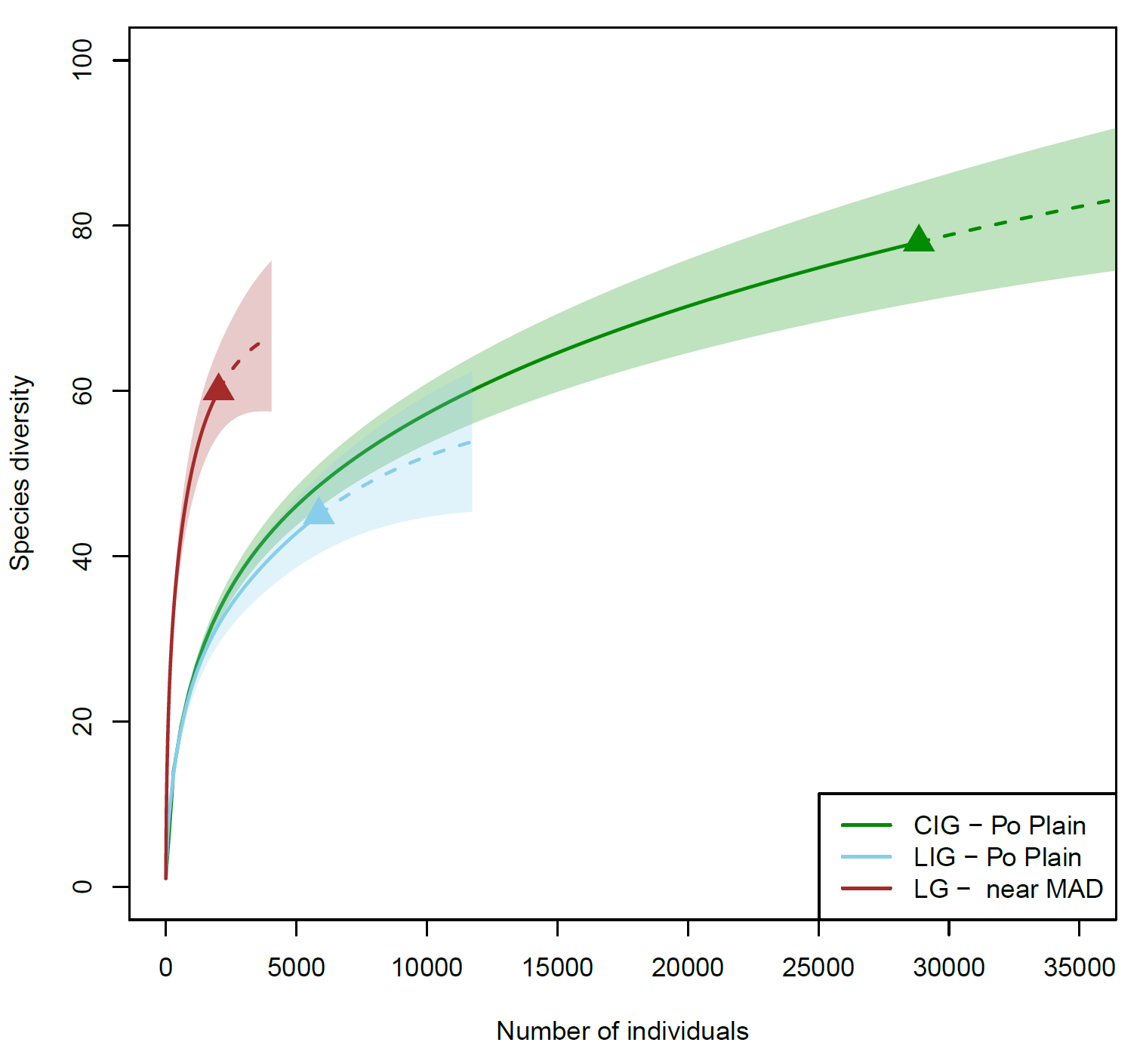


**Fig. S6.**

Rarefied species richness based on pooled data. Rarefaction (solid line) and extrapolation (dashed line) curves with 95% confidence intervals (shaded areas) based on species abundances pooled per each of the three-targeted intervals and calculated using iNEXT package (Hsieh et al., 2020). Abbreviations: LIG = last interglacial; LG = last late glacial, CIG = current interglacial.

| **Po-Adriatic dataset** | **Samples** | | | | |  | | **Species occurrence** | | | | |
| --- | --- | --- | --- | --- | --- | --- | --- | --- | --- | --- | --- | --- |
| **only nearshore samples** | **#** | **smallest** | **largest** | **mean** | **median** | | **total # of fossils** | | **total # of species** | **rarest** | **commonest** | **mean** |
| All samples (n>=0) | 223 | 0 | 16576 | 319.7 | 26.0 | 71282 | | 113 | | 1 | 174 | 10.8 |
| Samples with n>=1 s>=1 | 193 | 1 | 16576 | 369.3 | 46.0 | 71282 | | 113 | | 1 | 174 | 10.8 |
| Current Interglacial-CIG | 146 | 1 | 16576 | 386.9 | 34.0 | 56488 | | 78 | | 1 | 138 | 10.4 |
| Last late glacial-LG | 26 | 1 | 450 | 130.0 | 93.5 | 3381 | | 60 | | 1 | 20 | 4.2 |
| Last Interglacial-LIG | 21 | 4 | 5712 | 543.5 | 97.0 | 11413 | | 45 | | 1 | 16 | 3.7 |

**Table S1.**

Summary of the examined Adriatic sub-dataset used in this species-level study (that is, only gastropods, bivalves and scaphopods from nearshore samples considered cumulatively and within each climatic period investigated). The table, in addition to several sample descriptors, presents the original number of mollusk fossils identified at species-level (or from open nomenclature species when only one species of a targeted genus was retrieved), without accounting for disarticulation of bivalve shells (i.e. the abundance of bivalves is not divided by two). In Appendix S1 raw counts and stratigraphic information of the examined samples.

| **ID** | | **Well** | | **S-depth** | | **Age_ky** | | **Facies_integrated** | | **Ref_Age** | | **Ref_Facies** |
| --- | --- | --- | --- | --- | --- | --- | --- | --- | --- | --- | --- | --- |
| 677 | | S14 | | 3,05 | | <1.5 | | delta_front/beach_ridge | | Scarponi_et_2013-Geology | | Campo_et_al_2017-Palaeogeogr Palaeoclimatol Palaeoecol |
| 678 | | S14 | | 3,55 | | <1.5 | | delta_front/beach_ridge | | Scarponi_et_2013-Geology | | Campo_et_al_2017-Palaeogeogr Palaeoclimatol Palaeoecol |
| 679 | | S14 | | 3,98 | | <1.5 | | delta_front/beach_ridge | | Scarponi_et_2013-Geology | | Campo_et_al_2017-Palaeogeogr Palaeoclimatol Palaeoecol |
| 680 | | S14 | | 4,05 | | <1.5 | | delta_front/beach_ridge | | Scarponi_et_2013-Geology | | Campo_et_al_2017-Palaeogeogr Palaeoclimatol Palaeoecol |
| 681 | | S14 | | 4,55 | | <1.5 | | delta_front/beach_ridge | | Scarponi_et_2013-Geology | | Campo_et_al_2017-Palaeogeogr Palaeoclimatol Palaeoecol |
| 682 | | S14 | | 5,05 | | <1.5 | | delta_front/beach_ridge | | Scarponi_et_2013-Geology | | Campo_et_al_2017-Palaeogeogr Palaeoclimatol Palaeoecol |
| 683 | | S14 | | 5,25 | | <1.5 | | delta_front/beach_ridge | | Scarponi_et_2013-Geology | | Campo_et_al_2017-Palaeogeogr Palaeoclimatol Palaeoecol |
| 684 | | S14 | | 5,5 | | <1.5 | | delta_front/beach_ridge | | Scarponi_et_2013-Geology | | Campo_et_al_2017-Palaeogeogr Palaeoclimatol Palaeoecol |
| 629 | | S4 | | 8,55 | | <1.5 | | delta_front/beach_ridge | | Scarponi_et_2013-Geology | | Campo_et_al_2017-Palaeogeogr Palaeoclimatol Palaeoecol |
| 630 | | S4 | | 9,05 | | <1.5 | | delta_front/beach_ridge | | Scarponi_et_2013-Geology | | Campo_et_al_2017-Palaeogeogr Palaeoclimatol Palaeoecol |
| 631 | | S4 | | 9,55 | | <1.5 | | delta_front/beach_ridge | | Scarponi_et_2013-Geology | | Campo_et_al_2017-Palaeogeogr Palaeoclimatol Palaeoecol |
| 632 | | S4 | | 10,05 | | <1.5 | | delta_front/beach_ridge | | Scarponi_et_2013-Geology | | Campo_et_al_2017-Palaeogeogr Palaeoclimatol Palaeoecol |
| 1003 | | P2S | | 5,4 | | <3.0 | | beach_ridge | | Greggio_et_al_2017-Geol J | | Greggio_et_al_2017-Geol J |
| 1004 | | P2S | | 6,45 | | <3.0 | | beach_ridge | | Greggio_et_al_2017-Geol J | | Greggio_et_al_2017-Geol J |
| 685 | | S14 | | 6,05 | | <3.0 | | delta_front/beach_ridge | | Campo_et_al_2017-Palaeogeogr Palaeoclimatol Palaeoecol | | Campo_et_al_2017-Palaeogeogr Palaeoclimatol Palaeoecol |
| 687 | | S14 | | 7,05 | | <3.0 | | delta_front/beach_ridge | | Campo_et_al_2017-Palaeogeogr Palaeoclimatol Palaeoecol | | Campo_et_al_2017-Palaeogeogr Palaeoclimatol Palaeoecol |
| 688 | | S14 | | 7,55 | | <3.0 | | delta_front/beach_ridge | | Campo_et_al_2017-Palaeogeogr Palaeoclimatol Palaeoecol | | Campo_et_al_2017-Palaeogeogr Palaeoclimatol Palaeoecol |
| 689 | | S14 | | 8,05 | | <3.0 | | delta_front/beach_ridge | | Campo_et_al_2017-Palaeogeogr Palaeoclimatol Palaeoecol | | Campo_et_al_2017-Palaeogeogr Palaeoclimatol Palaeoecol |
| 690 | | S14 | | 8,55 | | <3.0 | | delta_front/beach_ridge | | Campo_et_al_2017-Palaeogeogr Palaeoclimatol Palaeoecol | | Campo_et_al_2017-Palaeogeogr Palaeoclimatol Palaeoecol |
| 691 | | S14 | | 9,05 | | <3.0 | | delta_front/beach_ridge | | Campo_et_al_2017-Palaeogeogr Palaeoclimatol Palaeoecol | | Campo_et_al_2017-Palaeogeogr Palaeoclimatol Palaeoecol |
| 692 | | S14 | | 9,13 | | <3.0 | | delta_front/beach_ridge | | Campo_et_al_2017-Palaeogeogr Palaeoclimatol Palaeoecol | | Campo_et_al_2017-Palaeogeogr Palaeoclimatol Palaeoecol |
| 693 | | S14 | | 9,55 | | <3.0 | | delta_front/beach_ridge | | Campo_et_al_2017-Palaeogeogr Palaeoclimatol Palaeoecol | | Campo_et_al_2017-Palaeogeogr Palaeoclimatol Palaeoecol |
| 696 | | S14 | | 11,05 | | <3.0 | | delta_front/beach_ridge | | Campo_et_al_2017-Palaeogeogr Palaeoclimatol Palaeoecol | | Campo_et_al_2017-Palaeogeogr Palaeoclimatol Palaeoecol |
| 634 | | S4 | | 11,05 | | <3.0 | | delta_front/beach_ridge | | Campo_et_al_2017-Palaeogeogr Palaeoclimatol Palaeoecol | | Campo_et_al_2017-Palaeogeogr Palaeoclimatol Palaeoecol |
| 795 | | S7 | | 6,45 | | <3.0 | | shoreface | | Amorosi_et_al_2017-Mar Pet Geo | | Amorosi_et_al_2003-Geol J |
| 799 | | S7 | | 8,95 | | <3.0 | | shoreface | | Amorosi_et_al_2017-Mar Pet Geo | | Amorosi_et_al_2003-Geol J |
| 806 | | S7 | | 12,45 | | <3.0 | | shoreface | | Amorosi_et_al_2017-Mar Pet Geo | | Amorosi_et_al_2003-Geol J |
| 586 | | S8 | | 3,8 | | <3.0 | | beach_ridge | | Campo_et_al_2017-Palaeogeogr Palaeoclimatol Palaeoecol | | Amorosi_et_al_2004-Global Planet Change |
| 587 | | S8 | | 4 | | <3.0 | | beach_ridge | | Campo_et_al_2017-Palaeogeogr Palaeoclimatol Palaeoecol | | Amorosi_et_al_2004-Global Planet Change |
| 588 | S8 | | 5,2 | | <3.0 | | beach_ridge | | Campo_et_al_2017-Palaeogeogr Palaeoclimatol Palaeoecol | | Amorosi_et_al_2004-Global Planet Change | |
| 593 | S8 | | 10,2 | | <3.0 | | beach_ridge | | Campo_et_al_2017-Palaeogeogr Palaeoclimatol Palaeoecol | | Amorosi_et_al_2004-Global Planet Change | |
| 594 | S8 | | 11,2 | | <3.0 | | beach_ridge | | Campo_et_al_2017-Palaeogeogr Palaeoclimatol Palaeoecol | | Amorosi_et_al_2004-Global Planet Change | |
| 526 | S10 | | 8 | | <3.5 | | beach_ridge | | Scarponi_et_2013-Geology | | Amorosi_et_al_2004-Global Planet Change | |
| 528 | S10 | | 9,4 | | <3.5 | | beach_ridge | | Scarponi_et_2013-Geology | | Amorosi_et_al_2004-Global Planet Change | |
| 529 | S10 | | 10 | | <3.5 | | beach_ridge | | Scarponi_et_2013-Geology | | Amorosi_et_al_2004-Global Planet Change | |
| 530 | S10 | | 12,3 | | <3.5 | | beach_ridge | | Scarponi_et_2013-Geology | | Amorosi_et_al_2004-Global Planet Change | |
| 532 | S10 | | 15 | | <3.5 | | beach_ridge | | Scarponi_et_2013-Geology | | Amorosi_et_al_2004-Global Planet Change | |
| 572 | S5 | | 12,08 | | <3.5 | | beach_ridge | | Scarponi_et_2013-Geology | | Amorosi_et_al_2004-Global Planet Change | |
| 573 | S5 | | 14,9 | | <3.5 | | beach_ridge | | Scarponi_et_2013-Geology | | Amorosi_et_al_2004-Global Planet Change | |
| 1098 | S2 | | 2,55 | | <6.0 | | shoreface | | Amorosi_et_al_2003-Geol J | | Amorosi_et_al_2003-Geol J | |
| 1099 | S2 | | 3,05 | | <6.0 | | shoreface | | Amorosi_et_al_2003-Geol J | | Amorosi_et_al_2003-Geol J | |
| 1100 | S2 | | 3,55 | | <6.0 | | shoreface | | Amorosi_et_al_2003-Geol J | | Amorosi_et_al_2003-Geol J | |
| 1101 | S2 | | 4,05 | | <6.0 | | shoreface | | Amorosi_et_al_2003-Geol J | | Amorosi_et_al_2003-Geol J | |
| 1102 | S2 | | 4,55 | | <6.0 | | shoreface | | Amorosi_et_al_2003-Geol J | | Amorosi_et_al_2003-Geol J | |
| 1103 | S2 | | 5,3 | | <6.0 | | shoreface | | Amorosi_et_al_2003-Geol J | | Amorosi_et_al_2003-Geol J | |
| 1104 | S2 | | 5,55 | | <6.0 | | shoreface | | Amorosi_et_al_2003-Geol J | | Amorosi_et_al_2003-Geol J | |
| 1105 | S2 | | 6,05 | | <6.0 | | shoreface | | Amorosi_et_al_2003-Geol J | | Amorosi_et_al_2003-Geol J | |
| 1107 | S2 | | 7,05 | | <6.0 | | shoreface | | Amorosi_et_al_2003-Geol J | | Amorosi_et_al_2003-Geol J | |
| 1109 | S2 | | 8,55 | | <6.0 | | shoreface | | Amorosi_et_al_2003-Geol J | | Amorosi_et_al_2003-Geol J | |
| 1112 | S2 | | 11,55 | | <6.0 | | shoreface | | Amorosi_et_al_2003-Geol J | | Amorosi_et_al_2003-Geol J | |
| 1113 | S2 | | 12,05 | | <6.0 | | shoreface | | Amorosi_et_al_2003-Geol J | | Amorosi_et_al_2003-Geol J | |
| 1114 | S2 | | 12,55 | | <6.0 | | shoreface | | Amorosi_et_al_2003-Geol J | | Amorosi_et_al_2003-Geol J | |
| 1115 | S2 | | 13,05 | | <6.0 | | shoreface | | Amorosi_et_al_2003-Geol J | | Amorosi_et_al_2003-Geol J | |
| 1507 | LSD-2-27 | | 5,54 | | 14.5-18.0 | | fluvial-influenced_nearshore | | Pellegrini_et_al_2018-Mar Pet Geo | | Gamberi_et_al_2020-Basin Res | |
| 1508 | LSD-2-27 | | 5,04 | | 14.5-18.0 | | fluvial-influenced_nearshore | | Pellegrini_et_al_2018-Mar Pet Geo | | Gamberi_et_al_2020-Basin Res | |
| 1434 | LSD-2-37 | | 0,79 | | 14.5-18.0 | | sandspit | | Pellegrini_et_al_2018-Mar Pet Geo | | Gamberi_et_al_2020-Basin Res | |
| 1435 | LSD-2-37 | | 0,43 | | 14.5-18.0 | | sandspit | | Pellegrini_et_al_2018-Mar Pet Geo | | Gamberi_et_al_2020-Basin Res | |
| 1630 | INV_12-08 | | 8,08 | | 15.0-18.0 | | fluvial-influenced_nearshore | | Maselli_et_al_2014-Quat Sci Rev | | Maselli_et_al_2014-Quat Sci Rev | |
| 1631 | INV_12-08 | | 7,76 | | 15.0-18.0 | | fluvial-influenced_nearshore | | Maselli_et_al_2014-Quat Sci Rev | | Maselli_et_al_2014-Quat Sci Rev | |
| 1632 | INV_12-08 | | 7,23 | | 15.0-18.0 | | fluvial-influenced_nearshore | | Maselli_et_al_2014-Quat Sci Rev | | Maselli_et_al_2014-Quat Sci Rev | |
| 1633 | INV_12-08 | | 6,83 | | 15.0-18.0 | | fluvial-influenced_nearshore | | Maselli_et_al_2014-Quat Sci Rev | | Maselli_et_al_2014-Quat Sci Rev | |
| 1634 | INV_12-08 | | 6,43 | | 15.0-18.0 | | fluvial-influenced_nearshore | | Maselli_et_al_2014-Quat Sci Rev | | Maselli_et_al_2014-Quat Sci Rev | |
| 1635 | INV_12-08 | | 6,24 | | 15.0-18.0 | | fluvial-influenced_nearshore | | Maselli_et_al_2014-Quat Sci Rev | | Maselli_et_al_2014-Quat Sci Rev | |
| 1636 | INV_12-08 | | 5,53 | | 15.0-18.0 | | fluvial-influenced_nearshore | | Maselli_et_al_2014-Quat Sci Rev | | Maselli_et_al_2014-Quat Sci Rev | |
| 1637 | INV_12-08 | | 4,63 | | 15.0-18.0 | | fluvial-influenced_nearshore | | Maselli_et_al_2014-Quat Sci Rev | | Maselli_et_al_2014-Quat Sci Rev | |
| 1639 | INV_12-08 | | 7,33 | | 15.0-18.0 | | fluvial-influenced_nearshore | | Maselli_et_al_2014-Quat Sci Rev | | Maselli_et_al_2014-Quat Sci Rev | |
| 1640 | MAP_12-01 | | 4,16 | | 15.0-18.0 | | fluvial-influenced_nearshore | | Maselli_et_al_2014-Quat Sci Rev | | Maselli_et_al_2014-Quat Sci Rev | |
| 1361 | LSD-2-26 | | 3,51 | | 15.5-13.5 | | fluvial-influenced_nearshore | | unpublished_^14^C_data | | Azzarone_et_al_2020-BSPI* | |
| 1364 | LSD-2-26 | | 2,2 | | 15.5-13.5 | | fluvial-influenced_nearshore | | unpublished_^14^C_data | | Azzarone_et_al_2020-BSPI* | |
| 1558 | LSD-2-26 | | 2,07 | | 15.5-13.5 | | fluvial-influenced_nearshore | | unpublished_^14^C_data | | Azzarone_et_al_2020-BSPI* | |
| 1365 | LSD-2-26 | | 1,21 | | 15.5-13.5 | | fluvial-influenced_nearshore | | unpublished_^14^C_data | | Azzarone_et_al_2020-BSPI* | |
| 1172 | S6 | | 6,58 | | 6.0-4.0 | | shoreface | | Amorosi_et_al_2017-Mar Pet Geo | | Amorosi_et_al_2003-Geol J | |
| 1173 | S6 | | 6,95 | | 6.0-4.0 | | shoreface | | Amorosi_et_al_2017-Mar Pet Geo | | Amorosi_et_al_2003-Geol J | |
| 1174 | S6 | | 7,45 | | 6.0-4.0 | | shoreface | | Amorosi_et_al_2017-Mar Pet Geo | | Amorosi_et_al_2003-Geol J | |
| 1175 | S6 | | 7,78 | | 6.0-4.0 | | shoreface | | Amorosi_et_al_2017-Mar Pet Geo | | Amorosi_et_al_2003-Geol J | |
| 1176 | S6 | | 8,33 | | 6.0-4.0 | | shoreface | | Amorosi_et_al_2017-Mar Pet Geo | | Amorosi_et_al_2003-Geol J | |
| 1177 | S6 | | 8,43 | | 6.0-4.0 | | shoreface | | Amorosi_et_al_2017-Mar Pet Geo | | Amorosi_et_al_2003-Geol J | |
| 1179 | S6 | | 10,33 | | 6.0-4.0 | | shoreface | | Amorosi_et_al_2017-Mar Pet Geo | | Amorosi_et_al_2003-Geol J | |
| 1180 | S6 | | 10,88 | | 6.0-4.0 | | shoreface | | Amorosi_et_al_2017-Mar Pet Geo | | Amorosi_et_al_2003-Geol J | |
| 1181 | S6 | | 11,93 | | 6.0-4.0 | | shoreface | | Amorosi_et_al_2017-Mar Pet Geo | | Amorosi_et_al_2003-Geol J | |
| 1182 | S6 | | 12,7 | | 6.0-4.0 | | shoreface | | Amorosi_et_al_2017-Mar Pet Geo | | Amorosi_et_al_2003-Geol J | |
| 1183 | S6 | | 13,05 | | 6.0-4.0 | | shoreface | | Amorosi_et_al_2017-Mar Pet Geo | | Amorosi_et_al_2003-Geol J | |
| 1184 | S6 | | 13,25 | | 6.0-4.0 | | shoreface | | Amorosi_et_al_2017-Mar Pet Geo | | Amorosi_et_al_2003-Geol J | |
| 549 | S10 | | 103,9 | | ~125-110 | | shoreface | | Scarponi_et_2004-Geology | | Scarponi_et_2013-Geology | |
| 550 | S10 | | 106,6 | | ~125-110 | | shoreface | | Scarponi_et_2004-Geology | | Calabrese & Cibin_2014-Geol Map | |
| 551 | S10 | | 107,35 | | ~125-110 | | shoreface | | Scarponi_et_2004-Geology | | Calabrese & Cibin_2014-Geol Map | |
| 894 | S2 | | 89,35 | | ~125-110 | | delta_front/beach_ridge | | Calabrese & Cibin_2014-Geol Map | | Calabrese & Cibin_2014-Geol Map | |
| 895 | S2 | | 90,9 | | ~125-110 | | delta_front/beach_ridge | | Calabrese & Cibin_2014-Geol Map | | Calabrese & Cibin_2014-Geol Map | |
| 897 | S2 | | 92,8 | | ~125-110 | | delta_front/beach_ridge | | Calabrese & Cibin_2014-Geol Map | | Calabrese & Cibin_2014-Geol Map | |
| 898 | S2 | | 94,35 | | ~125-110 | | delta_front/beach_ridge | | Calabrese & Cibin_2014-Geol Map | | Calabrese & Cibin_2014-Geol Map | |
| 899 | S2 | | 95,4 | | ~125-110 | | shoreface | | Calabrese & Cibin_2014-Geol Map | | Calabrese & Cibin_2014-Geol Map | |
| 1157 | S3 | | 79,85 | | ~125-110 | | beach_ridge | | Campo_et_al_2020-Quat Sci Rev | | Campo_et_al_2020-Quat Sci Rev | |
| 1158 | S3 | | 80,05 | | ~125-110 | | beach_ridge | | Campo_et_al_2020-Quat Sci Rev | | Campo_et_al_2020-Quat Sci Rev | |
| 1159 | S3 | | 80,55 | | ~125-110 | | beach_ridge | | Campo_et_al_2020-Quat Sci Rev | | Campo_et_al_2020-Quat Sci Rev | |
| 603 | S8 | | 121,3 | | ~125-110 | | beach_ridge | | Scarponi_et_2004-Geology | | Amorosi_et_al_2004-Global Planet Change | |
| 604 | S8 | | 123,7 | | ~125-110 | | beach_ridge | | Scarponi_et_2004-Geology | | Amorosi_et_al_2004-Global Planet Change | |
| 605 | S8 | | 124,4 | | ~125-110 | | beach_ridge | | Scarponi_et_2004-Geology | | Amorosi_et_al_2004-Global Planet Change | |
| 606 | S8 | | 125 | | ~125-110 | | beach_ridge | | Scarponi_et_2004-Geology | | Amorosi_et_al_2004-Global Planet Change | |
|  |  | |  | |  | |  | |  | |  | |

**Table S2.**

Previously published environmental and chronostratigraphic information concerning all nearshore samples used in multivariate analyses (i.e., containing at least 25 specimens, see Appendixes 1 and 2 for sample palaeontological and stratigraphic details). Such inferences were based on integrated sedimentological, lithological and micro and meiofossils data, hence largely independent from interpretations using macrofossil assemblages (only for 5 samples: 549, 1361-1365 environmental labels were retrieved from papers considering also macrobenthic data. Acronyms: ID = sample identification number; Well = core label; S-depth = sample core depth; Age_ky = inferred time interval of the sampled deposit, based on previously published papers or our unpublished data (see Ref_Age column). Unpublished age data are obtained by means of rapid carbonate-target radiocarbon dating method (Bright et al., 2020 and references therein); Facies_integrated and Ref_Facies = previously published sedimentary facies attribution of targeted samples.

Note: Papers cited in the table are included in the reference section.

| **NMDS1** | 4th-root | Log | Wisconsin |
| --- | --- | --- | --- |
| 4th-root | - | < 0.001 | < 0.001 |
| Log | 0.981 | - | < 0.001 |
| wisconsin | 0.827 | 0.802 | - |
| **NMDS2** | 4th-root | Log | Wisconsin |
| 4th-root | - | < 0.001 | < 0.001 |
| Log | 0.809 | - | < 0.001 |
| Wisconsin | 0.548 | 0.351 | - |

**Table S3.**

Correlation between sample scores in NMDS ordination based on different data transformations: relative 4th-root, log-transformation and Wisconsin double-relativisation. Results for NMDS axis 1 and axis 2 are shown separately with Pearson’s correlation coefficient in the lower triangle and *p*-values in the upper triangle of each table.

| **Species** | **Authorship** | **Holocene (CIG)**  Rank % abund. | | | **Biogeography** |  |
| --- | --- | --- | --- | --- | --- | --- |
| *Lentidium mediterraneum* | (O. G. Costa, 1830) | 1 | 0.885 | | MED/LUS |  |
| *Chamelea gallina* | (Linné, 1758) | 2 | 0.034 | | MED/LUS |  |
| *Donax semistriatus* | Poli, 1795 | 3 | 0.016 | | MED/LUS |  |
| *Spisula subtruncata* | (da Costa, 1778) | 4 | 0.012 | | COS |  |
| *Bittium reticulatum* | (da Costa, 1778) | 5 | 0.010 | | MED/LUS/BOR |  |
| *Varicorbula gibba* | (Olivi, 1792) | 6 | 0.009 | | COS |  |
| *Ecrobia ventrosa* complex*^1^* | (Montagu, 1803) | 7 | 0.005 | | COS |  |
| *Bela formica^2^* | (Risso, 1826) | 8 | 0.003 | | MED/LUS/BOR |  |
| *Peronidia albicans* | (Nordsieck, 1977) | 9 | 0.002 | | MED |  |
| *Tritia varicosa^3^* | (W. Turton, 1825) | 10 | 0.002 | | MED/LUS/BOR |  |
| **Species** | **Authorship** | **Pleistocene (LG)**  Rank % abund. | | | **Biogeography** |  |
| ***Spisula subtruncata*** | (da Costa, 1778) | 1 | 0.437 | | COS |  |
| *Rissoa monodonta* | Philippi, 1836 | 2 | 0.098 | | MED/LUS |  |
| ***Lentidium mediterraneum*** | (Linné, 1758) | 3 | 0.079 | | MED/LUS |  |
| *Abra alba* | (W.Wood, 1802) | 5 | 0.057 | | COS |  |
| ***Bittium reticulatum*** | (da Costa, 1778) | 4 | 0.056 | | MED/LUS/BOR |  |
| ***Ecrobia ventrosa* complex^1^** | (Montagu, 1803) | 6 | 0.041 | | COS |  |
| *Fabulina fabula* | (Gmelin, 1791) | 7 | 0.030 | | MED/LUS/BOR |  |
| *Pusillina lineolata* | (Michaud, 1830) | 8 | 0.027 | | MED/LUS |  |
| *Lucinella divaricata* | (Linné, 1758) | 9 | 0.014 | | MED/LUS/BOR |  |
| *Anomia ephippium* | Linné, 1758 | 10 | 0.013 | | COS |  |
|  |  |  |  |  |  |  |
|  |  |  |  |  |  |  |
| **Species** | **Authorship** | **Pleistocene (LIG)**  Rank % abund. | | | **Biogeography** |  |
| ***Lentidium mediterraneum*** | (Linné, 1758) | 1 | 0.857 | | MED/LUS |  |
| ***Spisula subtruncata*** | (da Costa, 1778) | 2 | 0.064 | | COS |  |
| *Turritellinella tricarinata* | (Brocchi, 1814) | 3 | 0.025 | | MED/LUS/BOR |  |
| *Abra renieri^4^* | (Bronn, 1831) | 4 | 0.009 | | MED/LUS |  |
| ***Chamelea gallina*** | (Linné, 1758) | 5 | 0.006 | | MED/LUS |  |
| ***Ecrobia ventrosa complex^1^*** | (Montagu, 1803) | 6 | 0.006 | | MED/LUS/BOR |  |
| ***Donax semistriatus*** | Poli, 1795 | 7 | 0.005 | | MED/LUS |  |
| ***Bittium reticulatum*** | (da Costa) | 8 | 0.003 | | MED/LUS/BOR |  |
| *Fabulina fabula* | (Gmelin, 1791) | 9 | 0.003 | | MED/LUS/BOR |  |
| ***Bela formica^2^*** | (Nordsieck, 1977) | 10 | 0.002 | | MED/LUS |  |
| **Specimen total abundance** | |  |  | |  |  |
| *Holocene interglacial* | | 28850 | | |  |  |
| *Pleistocene late glacial* | | 2025 | | |  |  |
| *Pleistocene last interglacial* | | 5867 | | |  |  |

**Table S4.**

Ten most abundant species in the three analysed intervals along with their biogeographic affinity (in bold most abundant species from LIG and LG, belonging also to the top-ten species of the CIG). As bivalves were commonly retrieved disarticulated, their abundances were divided by two (see Table S1 for a summary of total fossil remains at species level in each of the three climatic intervals). Abbreviations: MED/LUS/BOR = species occurring in the Mediterranean, Lusitanian and Boreal provinces; COS=Cosmopolitan; MED/LUS = species occurring in the Mediterranean and/or Lusitanian provinces. Taxonomic notes: 1 This is a group of very similar and highly variable species: *Ecrobia ventrosa*, *Hydrobia acuta* and *Eupaludestrina stagnorum* not easily distinguishable by the shell features (accessed through <http://marinespecies.org/aphia.php?p=taxdetails&id=1457275>); 2 *Bela formica* is considered taxon inquirendum previously synonymised with *Bela nebula* (accessed through <http://www.marinespecies.org/aphia.php?p=taxdetails&id=853246>); 3 commonly reported as *Tritia pygmaea* (Lamarck) a junior secondary homonym of *Muricites pygmaeus* Schlotheim (accessed through <http://www.marinespecies.org/aphia.php?p=taxdetails&id=876854>); 4 *A. renieri* is here considered an available species name (commonly synonymised with *A. alba* see <https://www.marinespecies.org/aphia.php?p=taxdetails&id=181518>).

| Pairwise | F. model | R^2^ | p value  (α=0.05) | p. adjusted  (Bonferroni correction) |
| --- | --- | --- | --- | --- |
| LG vs. CIG | 17.99 | 0.182 | 0.001 | 0.003 |
| LG vs. LIG | 5.81 | 0.158 | 0.001 | 0.003 |
| CIG vs. LIG | 1.69 | 0.021 | 0.144 | 0.432 |

**Table S5**.

Results of Permutational Multivariate Analysis of Variance (PERMANOVA) based on 999 permutations), point to strong differences in the centroid location and/or overall dispersion in the interglacial vs. glacial pairwise comparisons (LG vs. CIG and LG vs. LIG) and lack of significant differences between the two interglacial (CIG vs. LIG). As for glacial vs. interglacial comparisons, significant differences in the location and/or dispersion persist also when the strict Bonferroni correction is applied. Although PERMANOVA is widely used for testing multivariate differences among groups, it tends to be influenced by heterogeneity derived from unbalanced sampling. Given the strong differences in the number of samples from the three time intervals, here we apply PERMANOVA comparatively and not as a formal statistical test. PERMANOVA (and NMDS) output all consistently suggest a greater multivariate similarity between the two interglacial groups of nearshore samples with respect to those between interglacial and glacial assemblages. PERMANOVA is based on the dataset restricted to samples with ≥ 25 specimens.

| **Total**  **Dataset**  **(species abundance)** | **Param** | **CIG-LG** | **CIG-LIG** | **LG-LIG** | **CIG-LG no 0s** | **CIG-LIG no 0s** | **LG-LIG no 0s** |
| --- | --- | --- | --- | --- | --- | --- | --- |
|  | ρ | 0.019 | 0.512 | 0.033 | -0.038 | 0.437 | -0.159 |
|  | p | 0.842 | **0.000** | 0.731 | 0.697 | **0.000** | 0.142 |

**Table S6**.

Spearman’s rank correlations. Only the interglacial pairwise comparisons (CIG-LIG columns) report consistently significant and positive ρ values when total species abundance is considered. In addition, when species absent in both intervals are excluded (“no 0s” columns) pairwise comparisons highlight consistently significant positive ρ values). All comparisons between the glacial and interglacial assemblages are not significant.

**References**

Amorosi A., Barbieri G., Bruno L., Campo B., Drexler T.M., Hong W., Rossi V., Sammartino I., Scarponi D., Vaiani S.C., Bohacs K.M. 2019. Three-fold nature of coastal progradation during the Holocene eustatic highstand, Po Plain, Italy – close correspondence of stratal character with distribution patterns. Sedimentology, 7, 3029–3052.

Amorosi A., Bruno L., Campo B., Costagli B., Dinelli E., Hong W., Sammartino I., Vaiani S.C. 2020. Tracing clinothem geometry and sediment pathways in the prograding Holocene Po Delta system through integrated core stratigraphy. Basin Res. 32.

Amorosi A., Bruno L., Campo B., Morelli A., Rossi V., Scarponi D., Hong W., Bohacs K.M., Drexler T.M. 2017. Global sea-level control on local parasequence architecture from the Holocene record of the Po Plain, Italy. Mar. Petrol. Geol. 87, 99–111.

Amorosi A., Centineo M C., Colalongo M L., Pasini G., Sarti G., Vaiani S.C. 2003. Facies architecture and Latest Pleistocene-Holocene depositional history of the Po Delta (Comacchio area), Italy. J. Geol. 111, 39–56.

Amorosi A., Colalongo M., Fiorini F., Fusco F., Pasini G., Vaiani S., Sarti G. 2004. Palaeogeographic and palaeoclimatic evolution of the Po Plain from 150-ky core records. Global Planet. Change 40, 55–78.

Artegiani A., Paschini E., Russo A., Bregant D., Raicich F., Pinardi N. 1997. The Adriatic Sea General Circulation. Part I: Air–Sea Interactions and Water Mass Structure. J. Phys. Oceanogr. 27, 1492–1514.

Azzarone M., Pellegrini C., Barbieri G., Rossi V., Gamberi F., Trincardi F., Scarponi D. 2020. Linking benthic fauna and seismic facies to improve stratigraphic reconstructions: the case of the Mid-Adriatic Deep since the late glacial period (Central Adriatic Sea). Boll. Soc. Pal. 59, 9–23.

Bright J., Ebert C., Kosnik M.A., Southon J.R., Whitacre K., et al.. 2021. Comparing direct carbonate and standard graphite 14C determinations from an assortment of biogenic carbonates. Radiocarbon, 63(2), 387–403

Bruno L., Bohacs K.M., Campo B., Drexler T.M., Rossi V., Sammartino I., Scarponi D., Hong W., Amorosi A. 2017. Early Holocene transgressive palaeogeography in the Po coastal plain (northern Italy). Sedimentology, 64, 1792–1816.

Calabrese L., Cibin U. 2014. Note illustrative della Carta Geologica d’Italia alla scala 1:50.000. Foglio 222 - Lugo. Regione Emilia Romagna. Servizio Geologico, Sismico e dei Suoli, 182 pp.

Campo B., Amorosi A., Vaiani S.C. 2017. Sequence stratigraphy and late Quaternary paleoenvironmental evolution of the Northern Adriatic coastal plain (Italy). Palaeogeogr. Palaeoclimatol. Palaeoecol. 466, 265–278.

Campo B., Bruno L., Amorosi A. 2020. Basin-scale stratigraphic correlation of late Pleistocene-Holocene (MIS 5e-MIS 1) strata across the rapidly subsiding Po Basin (northern Italy). Quaternary Science Reviews, 237, 106300.

Correggiari A., Cattaneo A., Trincardi F. 2005. The modern Po Delta system: Lobe switching and asymmetric prodelta growth. Mar. Geol. 222–223, 49–74.

Gamberi F., Pellegrini C., Dalla Valle G., Scarponi D., Bohacs K., Trincardi F. 2020. Compound and hybrid clinothems of the last lowstand Mid‐Adriatic Deep: Processes, depositional environments, controls and implications for stratigraphic analysis of prograding systems. Basin Res. 32, 363–377.

Greggio N., Giambastiani B.M.S., Campo B., Dinelli E., Amorosi A. 2017. Sediment composition, provenance, and Holocene paleoenvironmental evolution of the Southern Po River coastal plain (Italy). Geol J. 53, 914–928.

Hsieh T.C., Ma K.H., Chao A. 2020. iNEXT: iNterpolation and EXTrapolation for species diversity. R package version 2.0.20 URL: http://chao.stat.nthu.edu.tw/wordpress/software-download/

Maselli V., Trincardi F., Asioli A., Ceregato A., Rizzetto F., Taviani M. 2014. Delta growth and river valleys: the influence of climate and sea level changes on the South Adriatic shelf (Mediterranean Sea). Quat. Sci. Rev. 99, 146–163.

Pellegrini C., Asioli A., Bohacs K.M., Drexler T.M., Feldman H.R., Sweet M.L., Maselli V., Rovere M., Gamberi F., Della Valle G., Trincardi F. 2018. The late Pleistocene Po River lowstand wedge in the Adriatic Sea: Controls on architecture variability and sediment partitioning. Mar. Petrol. Geol. 96, 16–50.

Pellegrini C., Maselli V., Cattaneo A., Piva A., Ceregato A., Trincardi F. 2015. Anatomy of a compound delta from the post-glacial transgressive record in the Adriatic Sea. Mar. Geol., 362, 43–59.

Pellegrini C., Maselli V., Trincardi F. 2016. Pliocene–Quaternary contourite depositional system along the south-western Adriatic margin: changes in sedimentary stacking pattern and associated bottom currents. Geo-Marine Letters, 36, 67–79.

Ridente D, Foglini F, Minisini D, Trincardi F, Verdicchio G. 2007. Shelf-edge erosion, sediment failure and inception of Bari Canyon on the Southwestern Adriatic Margin (Central Mediterranean). Mar. Geol. 246, 193–207.

Scarponi D., Kowalewski M. 2004. Stratigraphic paleoecology: Bathymetric signatures and sequence overprint of mollusk associations from upper Quaternary sequences of the Po Plain, Italy. Geology, 32, 989.

Sheppard T.H. 2006. Sequence architecture of ancient rocky shorelines and their response to sea level change: an Early Jurassic example from South Wales, UK. Geol Soc. J. 163, 595–606.
